# Supplementary material for: T-bet+ B cells are activated by and control endogenous retroviruses through TLR-dependent mechanisms
Source: Nat Commun. 2024 Feb 9;15:1229. doi: 10.1038/s41467-024-45201-6 (PMC10858178; doi:10.1038/s41467-024-45201-6)
Supplement: Supplementary file 1 — Supplementary Information [file 41467_2024_45201_MOESM1_ESM.pdf]

Supplementary Information

T-bet$^+$ B cells are activated by and control endogenous retroviruses through TLR-dependent mechanisms

Eileen Rauch et al.

Supplementary Table 1

Reagent/Resource	Source	Identifier
**Antibodies**		
anti-CD4 PE (Dil.: 1:300; clone GK1.5)	Thermo Fisher Scientific	A18667
anti-CD8a APC (Dil.: 1:300; clone 53-6.7)	BD Biosciences	553035
anti-CD19 PE (Dil.: 1:300; clone eBio1D3)	Thermo Fisher Scientific	12-0193-82
anti-Ly-6G PE (Dil.: 1:300; clone 1A8)	Thermo Fisher Scientific	12-9668-82
anti-CD45R/B220 APC (Dil.: 1:300; RA3-6B2)	BD Biosciences	553092
anti-CD23 PE (Dil.: 1:300; clone B3B4)	BD Pharmingen	553139
anti-CD23-Biotin (Dil.: 1:300; clone B3B4)	Biolegend	101604
anti-CD23 APC (Dil.: 1:300; clone B3B4)	Thermo Fisher Scientific	10725333
anti-CD11c PE (Dil.: 1:300; clone N418)	Thermo Fisher Scientific	MCD 11C04
anti-CD90.2 APC (Dil.: 1:300; clone 53-2.1)	BD Biosciences	533007
anti-IgE-PE (Dil.: 1:300; clone 23G3)	eBioscience	12-5992-83
anti-IgE-FITC (Dil.: 1:300; clone 23G3),	eBioscience	11-5992-81
anti-CD20-Biotin (Dil.: 1:300; clone SA275A11)	Biolegend	150414
anti-IgD-Biotin (Dil.: 1:300; clone 11-26c)	Invitrogen	13-5993-82
anti-IgD-FITC (Dil.: 1:750; clone 11-26c)	Invitrogen	MIGD01
anti-IgM-APC (Dil.: 1:300; clone II/41)	BD Biosciences	550676
anti-GL7-PE (Dil.: 1:300; clone GL-7)	eBioscience	12-5902-82
anti-CD19-PE (Dil.: 1:300; clone eBio1D3)	eBioscience	12-0193-8
anti-CD24-FITC (Dil.: 1:300; clone M1/69)	Invitrogen	11-0242-85
anti-T-bet-e660 (Dil.: 1:300; clone eBio4B10)	eBioscience	A18559
anti-CD80-PE (Dil.: 1:300; B7-19)	eBiosciences	12-0801-82
anti-CD73-APC (Dil.: 1:300; clone TY/11.8)	BD Pharmingen	567499
anti-IgM-FITC (Dil.: 1:300)	Southern Biotechnology Assoc.	1140-02
anti-IgG (H+L) Alexa Fluor 633 ab(IH-Dil.: 1:100)	Thermo Fisher Scientific	A-21050
anti-CD21/CD35 PE (IH-Dil.: 1:25; clone 7E9)	Miltenyi Biotec	130-102-440
anti-CD23 APC (IH-Dil.: 1:300; clone B3B4)	Thermo Fisher Scientific	# MCD 2305
anti-F4/80 PE (IH-Dil.: 1:100; clone BM8)	Thermo Fisher Scientific	# MF 48004
anti-CD4 APC (IH-Dil.: 1:100; clone RM4-5)	BD Biosciences	553051
goat anti-IgM (Endconc.: 25μg/ml)	BioRad	STAR86
anti-mouse-IgG (H+L) HRP (Dil.: 1:5000)	Jackson Immuno Research	115-035-166
anti-IgE (Endconc.: 10μg/ml; clone R35-72)	fisher scientific	10628810
goat anti-IgE-HRP (Dil.: 1:5000)	Southern Biotech	1110-05
mouse IgE (clone MEA-36)	BioLegend	401701
goat anti-mouse IgG-HRP (Dil.: 1:5000)	Thermo Fisher Scientific	31431
ChromPure rat IgG who.mol. (Dil.: 1:75)	Jackson Immuno Research	012-000-003
CD11c APC-cy7 (Dil.: 1:300; clone HL3)	BD Bioscience	581241
CD11b BV 785 (Dil.: 1:300; M1/70)	BioLegend	101243
CD21 BV711 (Dil.: 1:300; 7E9)	BioLegend	123435
CD23 bio (Dil.: 1:300; B3B4)	BD Bioscience	553137
GL7 perCP Cy5.5 (Dil.: 1:300; GL7)	BioLegend	144610
T-bet APC (Dil.: 1:300; 4B10)	BioLegend	644814
CD19 APC cy7 A5 (Dil.: 1:300; 6D5)	Biolegend	115529
Strept BV570 (Dil.: 1:1000)	BioLegend	405227
Strept BV510 (Dil.: 1:1000)	BioLegend	405234
Pcx Ab (Dil.: 1:1000)	ThermoFischer	PA5-72953
**Chemicals, peptides, kits and recombinant proteins**		
calf thymus DNA	Invitrogen	15633019
SuperSignal West Dura cl substrate	Thermo Fisher Scientific	34075
ELISpot substrate AEC	Sigma-Aldrich/Merck	152226BD CBA
Mouse Inflammation Kit	BD Biosciences	552364
Streptavidin-APC	Thermo Fisher Scientific	SA1005
pJET-2.1 vector	Thermo Fisher	K1231
peqGOLD Total RNA Kit	VWR	13-6834-01P
RevertAid First Strand cDNA Kit	Thermo Fisher	K1631
CD43 microbeads	Miltenyi	130-049-801

EXTRAzol	Blirt S.A.	EM30
ChloroformReagentPlus	Sigma-Aldrich	132950-1L
QuantiTect Reverse Transcription Kit	QIAGEN	205311
PowerUp SYBR Green Master Mix	Applied Biosystems	A25776
RNase-Free water	Thermo Fisher	10977035
QUANTI-Blue Solution	InvivoGen	rep-qbs2
Reporter Lysis Buffer	Promega	E3971
LPS E.Coli O127:B8	Sigma-Aldrich	297-473-0
R848	InvivoGen	tlrl-r848
Pam3Cysk4	InvivoGen	tlrl-pms
Ibrutinib	Selleckchem	S2680
Propidium Iodide	BioLegend	421301
CellTrace Far Red	Thermo Fischer	C334572
Streptavidin-PerCP	BioLegend	405213
peanut agglutinin Alexa Fluor 647	Molecular Probes	L32460
DAPI	Thermo Fisher Scientific	62248
Mowiol	Roth	0713.1
Fluo-4	Molecular Probes	F14217
HBSS	PAN Biotech	P04-49505
GFP	Abcam	ab84191o-
Phenylenediamine dihydrochloride	Sigmaaldrich/Merck	AEC101-1KT
Th1/Th2/Th17 CBA Kit	Becton Dickinson	AB_2869354

Oligonucleotides

1668 PTO-ODN	TIB MOLBIOL, Berlin	custom order

hA3Tg 5´-GGCACACAATGCCACACACTATGGCCTTCAGG-3´

5´-GTGCCCAGCATGTGTGCCATGGCTCAAGTTTG-3´

ERV-GFP insertion Chr.19
1694 5´-AACAGCTCCCACCTAGACAC-3´
1695 5´-GGAGACCCAGGGCTGTTAAT

ERV-GFP
1384 5´-ACAACAATCTCACCTCTGACCA-3´
1385 5´-AAGTCGTGCTGCTTCATGTG-3´

Q-PCR
actin, reference gene
5'-CTACAATGAGCTGCGTGTGG-3'
5'-CAAGCTCACACTTCATGATGG-3'

ERV-GFP 5'-ACAACAATCTCACCTCTGACCA-3'
5'-AAGTCGTGCTGCTTCATGTG-3'

MuLV

687 MLV1a 5' GGAGGGGTACGTGGTTCTTT 3'

688 MLV1b 5'GCTGGACATCTTCCCAGTGT 3'

ERV-GFP

1386-pMOV-GFP FW 5'TATTCGGTTTACAGACGCCG3'

1387 pMOV-GFP R	5’CGTAGGTCAGGGTGGTCAC 3‘

IFNg

1744 IFNg qPCR-FW	5‘CAGCAACAGCAAGGCGAAAAAGG3‘
1745 IFNg mouse qPCR-R	5’TTTCCGCTTCCTGAGGCTGGAT3‘

STAT-1

1750 mStat1-1FP	5’GCCTCTCATTGTCACCGAAGAAC3‘
1751 mStat1-RP	5’TGGCTGACGTTGGAGATCACCA3‘

T-bet

1760 mT-bet FP	5’CAACAACCCCTTTGCCAAAG3‘
1761 mT-bet RP	5’TCCCCCAAGCAGTTGACAGT3‘

Pcx

pcx-a	5´TAGACATCAAGGTGGCAGCA3´
pcx-b	5´TGTTGAGAGGCTTGGGGTAG3´
pcx-1	5´GAACATCCGCATCAATGGCT3´
pcx-2	5´CAGGAAGGGGATGTTGGTCT3´

AID

1754 mAID FP	5’TCTGCTACGTGGTGAAGAGGAG3‘
1755 mAID RP	5’CCAGTCTGAGATGTAGCGTAGG3‘

Software

FlowJo	https://www.bdbiosciences.com/en-us/ products/software/flowjo-v10-software	Version 9
GraphPad Prism	https://www.graphstats.net	Version 9.0
Bruker MI SE software	Bruker	
FIJI	https://imagej.net/software/fiji/downloads	
Primer3 software	https://primer3.ut.ee	Version4.1.0

Mouse strains

C57BL/6	The Jackson Laboratory
hA3 transgenic mice[1]	
EGT-315 B6	this study
EZGT-332/3	this study
Tlr7-deficient[2]	

$Tlr3^{-/-}Tlr7^{-/-}Tlr9^{-/-}$ triple deficient[3]		
T-bet$^{-/-}$ (tbx21)	The Jackson Laboratory	004648

Cell lines

WEHI-231	ATCC	CRL-1702
NIH-3T3	ATCC	CRL-1658
MEF	mouse embryonic fibroblasts	C57BL/6
HEK-Blue hTLR7 Cells	InvivoGen	
HEK mTlr9 cells[4]		
HEp-2 cells	ATCC	CCL-23
V6.5f1(C57BL/6 x 129/Sv)[5]	Thomas Wunderlich, Max Planck Institute for Metabolism Research, Cologne, Germany	
SP2/0-Ag14	ATCC	
40LB	Dai Kitamura, Tokyo, Japan	

Supplementary Table 2

Inheritance of ERV-GFP

Total mice: 898 (100%)	EGT-315 B6<br>453 mice (50.4%)	wildtype<br>445 (49.6%)
Total mice: 576 (100%)	EGT-315 $Tlr3^{-/-}Tlr7^{-/-}Tlr9^{-/-}$<br>264 mice (45.8%)	$Tlr3^{-/-}Tlr7^{-/-}Tlr9^{-/-}$<br>312 mice (54.2%)

Mating of heterozygous male EGT-315 with either C57BL/6 or $Tlr3^{-/-}Tlr7^{-/-}Tlr9^{-/-}$ female mice demonstrates a close to expected mendelian frequency for EGT-315 B6 and slight suppression for EGT-315 $Tlr3^{-/-}Tlr7^{-/-}Tlr9^{-/-}$ offspring. Importantly, this supports the data that a stable single autosomal insertion occurred and is inherited.

Supplementary Table 3

Chromosome (Chr.) location of split reads with ERV-GFP Integration. Cov.: Respective split coverage in bp, Str.: Strand. Reads are deposited at NCBI SRA, see Data and Materials availability.

**Chromosomal split**				**PMOV-GFP split**			
**Chr.**	**Position**	**Str.**	**Cov.**	**Position**	**Str.**	**Cov.**	**Read ID**
13	116,835,161	+	70	8,846	-	22	2211:19479:31845
19	4,666,860	-	109	38	+	39	2138:24731:11068
19	4,666,865	-	104	31	+	46	1627:18195:20885
19	4,666,877	-	92	19	+	58	2302:14380:15687

19	4,666,880	+	89	17	-	60	2371:10474:23328
19	4,666,880	+	89	17	-	60	2371:10646:22091
19	4,666,916	-	53	1	+	76	2644:12454:15076
19	4,666,921	-	48	1	+	76	1478:20880:5431
19	4,666,934	-	35	1	+	76	1320:26955:21151
19	4,666,965	+	107	8,456	-	43	1304:2157:22075
19	4,666,965	-	112	8,456	+	37	1510:18539:11804
19	4,666,965	-	112	8,456	+	37	1510:18656:11725
19	4,666,965	-	121	8,456	+	27	1244:9588:30968
19	4,666,965	+	122	8,456	-	28	1170:17246:22592
19	4,666,965	+	22	8,456	-	128	1276:20365:24925
19	4,666,965	-	30	8,456	+	118	1610:4788:31767
19	4,666,965	-	53	8,456	+	92	2307:18701:3693
19	4,666,965	+	57	8,456	-	93	1674:9245:26240
19	4,666,965	-	73	8,456	+	77	2275:4643:32111
19	4,666,965	+	90	8,456	-	56	2246:13503:31422

Supplementary Fig. S1

Rauch et al.

a

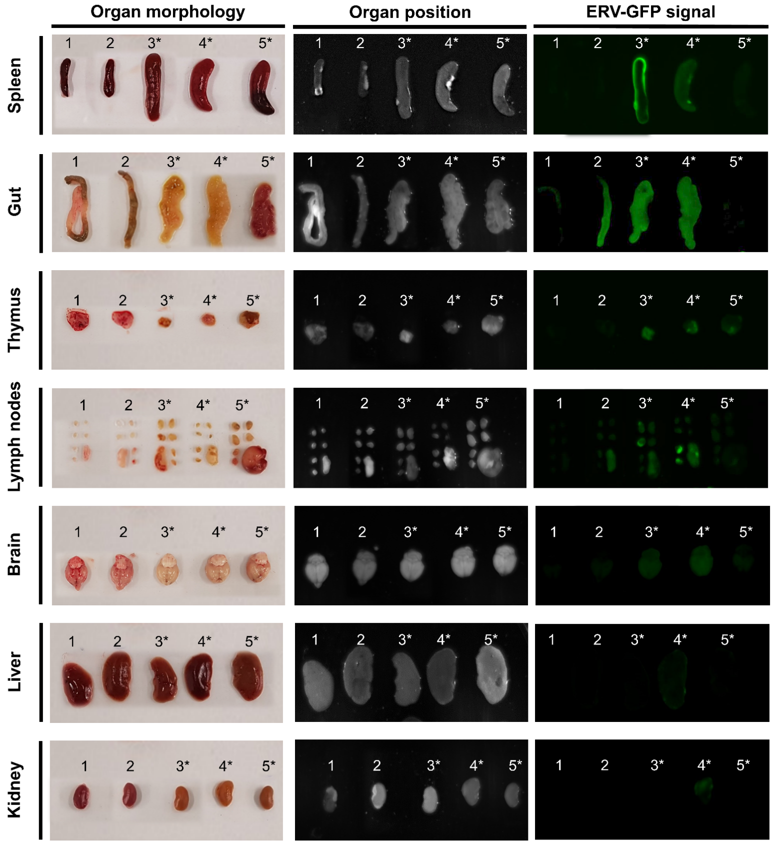

b

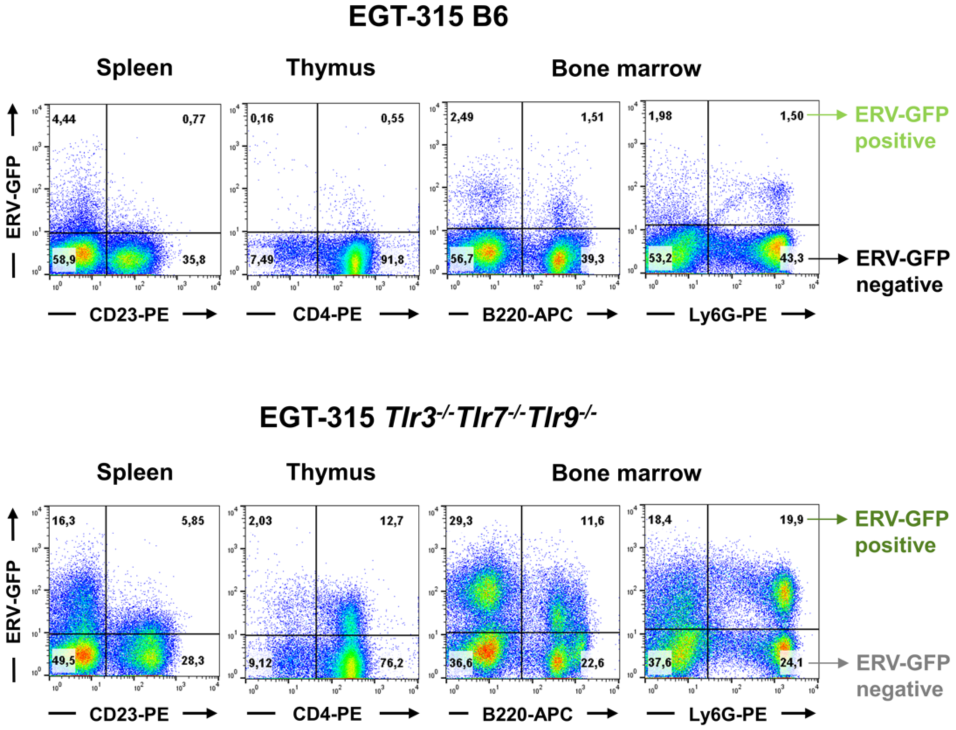

c

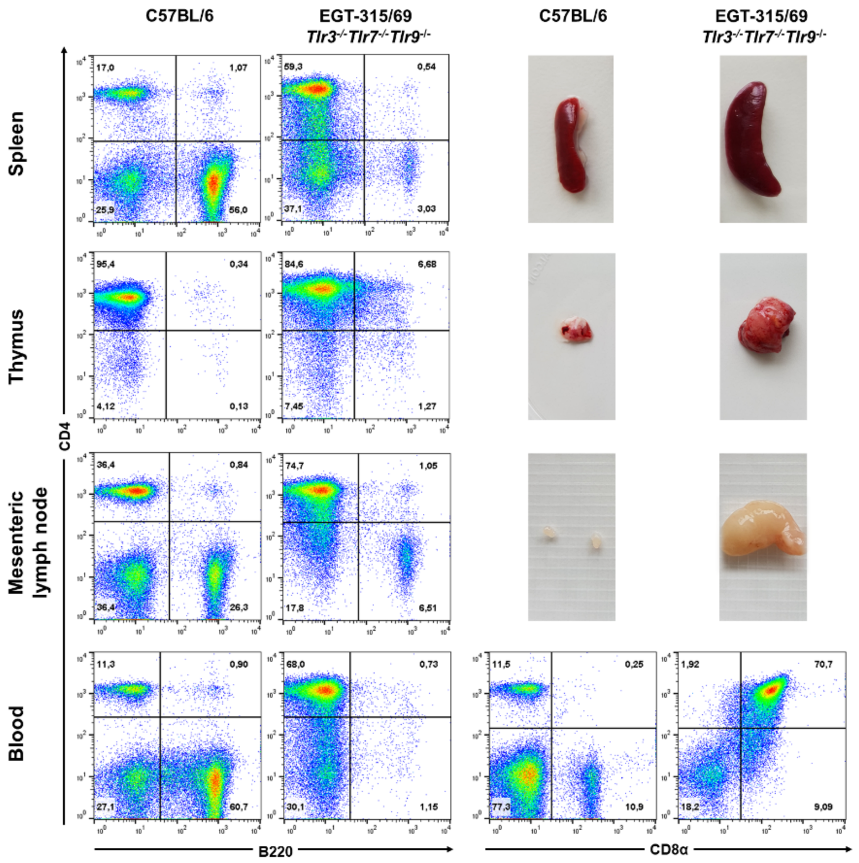

d

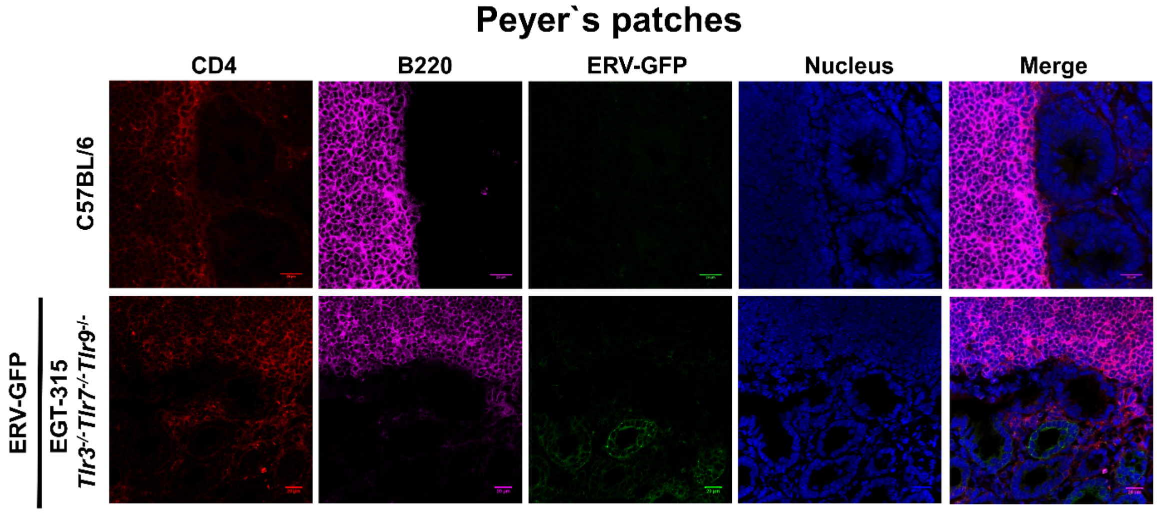

Figure S1 for Fig 1. ***Expression of ERV-GFP in different organs and T cell leukemia formation in EGT-315 mice***

a, *Ex vivo* imaging of the ERV-GFP signal from organs of EGT-315 B6 (n=1) and EGT-315 Tlr3$^{-/-}$Tlr7$^{-/-}$Tlr9$^{-/-}$ mice (n=3) with the Xtreme BI 4MP-X-ray (Bruker Bio Spin MRI). Mice 3-5 show T cell leukemia. We previously showed that the dominant adverse effect of ERV reactivation in Tlr3$^{-/-}$Tlr7$^{-/-}$Tlr9$^{-/-}$ mice is the development of *late*

onset T cell leukemia, with a mean survival of 85 weeks[3]. EGT-315 $Tlr3^{-/-}Tlr7^{-/-}Tlr9^{-/-}$ mice succumbed to *early* onset T cell leukemia with a mean survival of 21 weeks (SD= 5.9 weeks; n= 10). This suggests that the replication competent ERV is causing a higher rate of *early* onset T cell leukemia probably due to increased insertional mutagenesis in oncogenes. Genotypes: all mice mice tested were EGT-315 heterozygote and: 1 littermate control; 2, 4 and 5 are $Tlr3^{-/-}Tlr7^{-/-}Tlr9^{-/-}$; 3 is $Tlr3^{-/-}Tlr7^{-/-}Tlr9^{+/-}$. * marks mice with leukemia. **b**, Cell type-specific expression of ERV-GFP in lymphoid and myeloid cells of different organs. Representative examples for EGT-315 B6 mice (F2-F4, n= 5) and EGT-315-$Tlr3^{-/-}Tlr7^{-/-}Tlr9^{-/-}$ mice (F2-4, n= 5). Percentages are relative to the live cell gate. B cells (B220); mature B cells (CD23), Neutrophils (Ly6G), Dendritic cells (CD11c), T helper cells (CD4) and cytotoxic T cells (CD8). **c**, T cell leukemia formation, enlarged lymphoid organs and disturbed lymphocyte populations in EGT-315-$Tlr3^{-/-}Tlr7^{-/-}Tlr9^{-/-}$ mice. Exemplary flow cytometry of different lymphoid organs using anti-CD4, anti-CD8 and anti-CD45R/B220 staining. **d**, Confocal microphotograph of Peyer´s patches of 2.5-month old mice generations F1-4 with signals for CD4, B220, DAPI and ERV-GFP. Representative for one C57BL/6 mouse and four EGT-315 $Tlr3^{-/-}Tlr7^{-/-}Tlr9^{-/-}$ mice. Scale bar 25 µm.

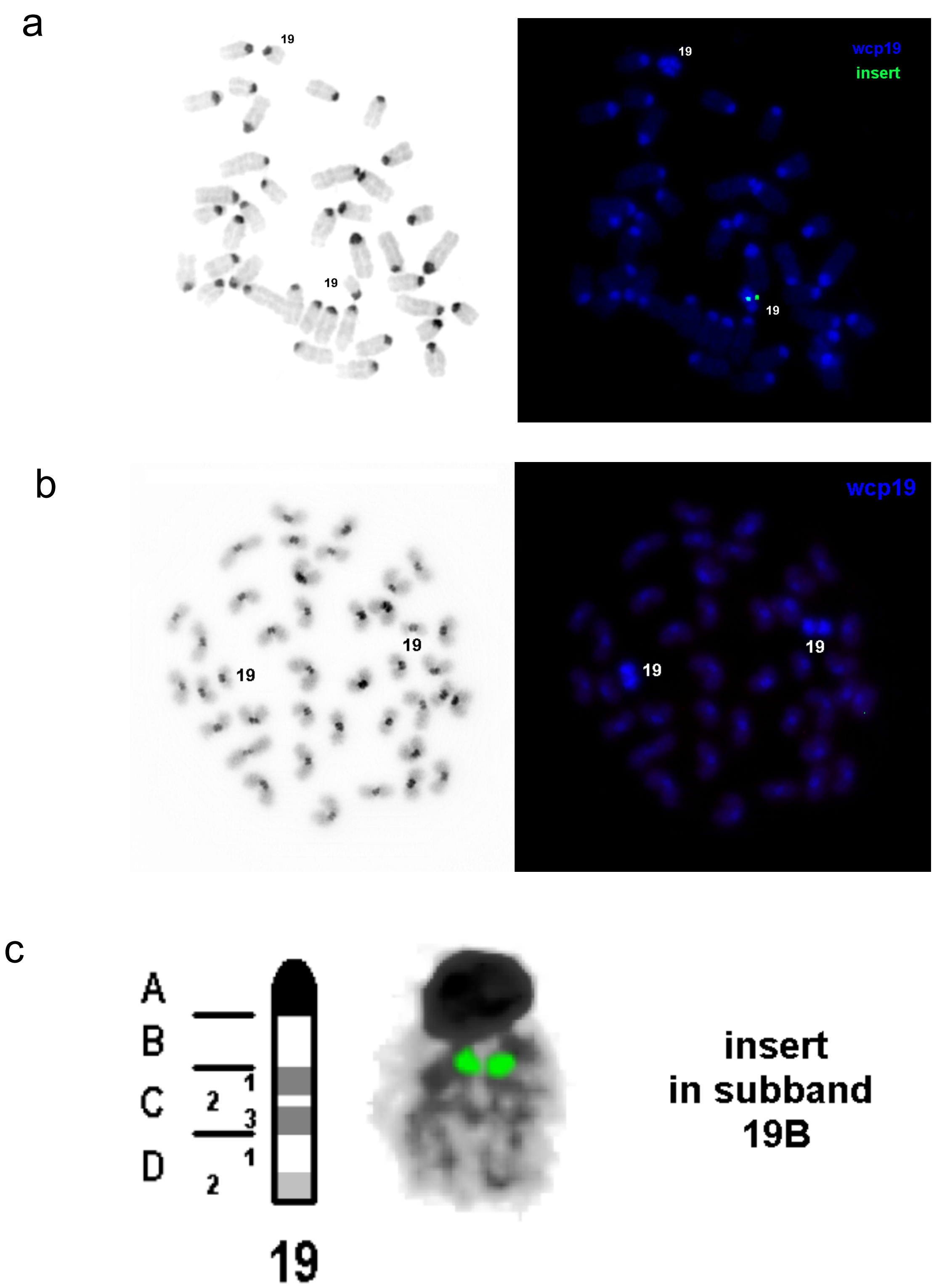
a
19
19
19
wcp19
insert
19
b
19
19
wcp19
19
19
c
A
B
C
D
2
1
3
1
2
19
insert
in subband
19B

d

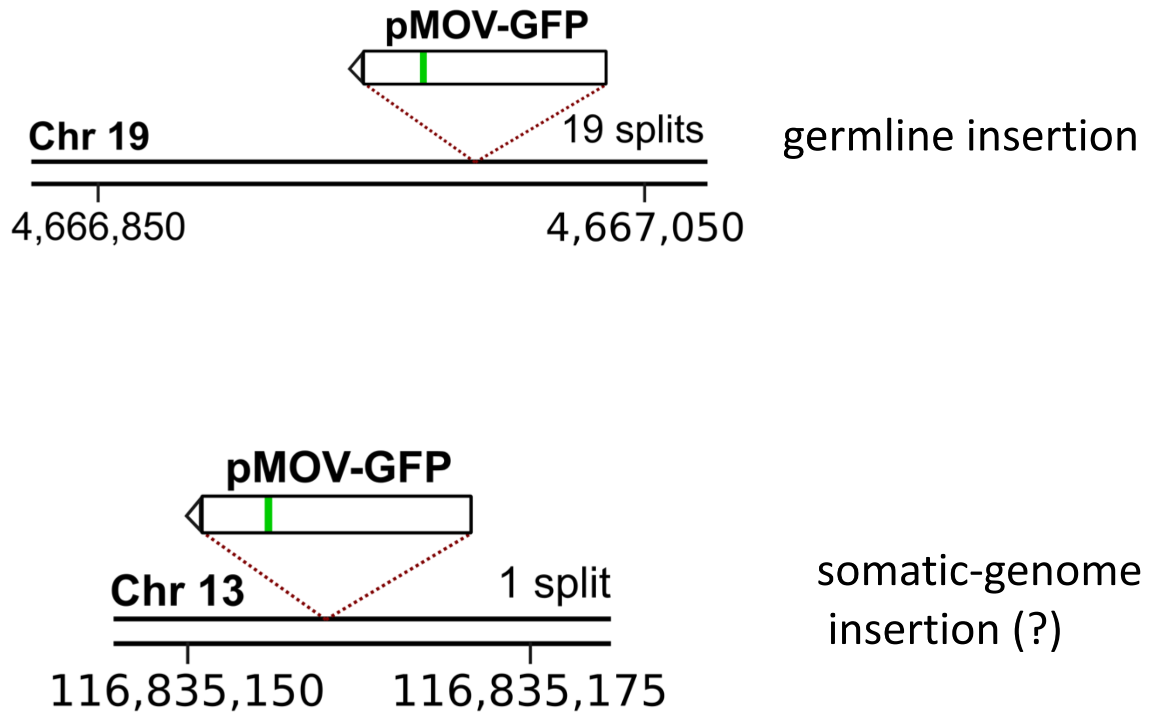

e

Pcx large intron

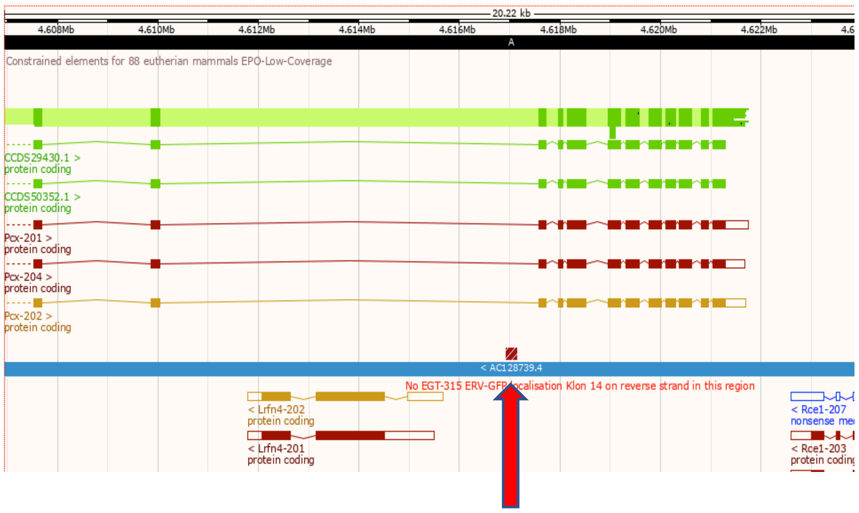

ERV-GFP Insertion

f

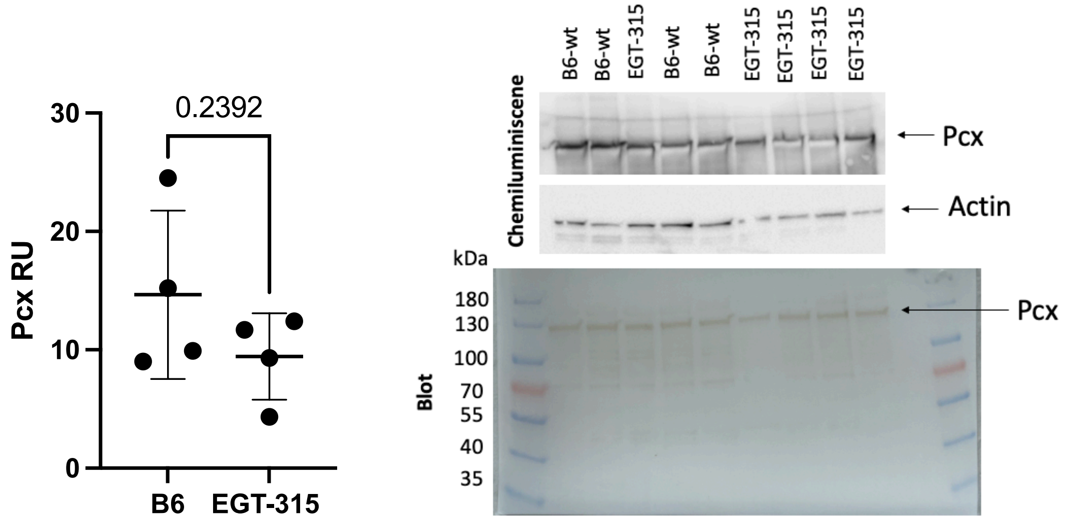
0.2392
Pcx RU
30
20
10
0
B6
EGT-315
B6-wt
B6-wt
EGT-315
B6-wt
B6-wt
EGT-315
EGT-315
EGT-315
EGT-315
Chemiluminiscene
Pcx
Actin
kDa
180
130
100
70
55
40
35
Blot
Pcx

g

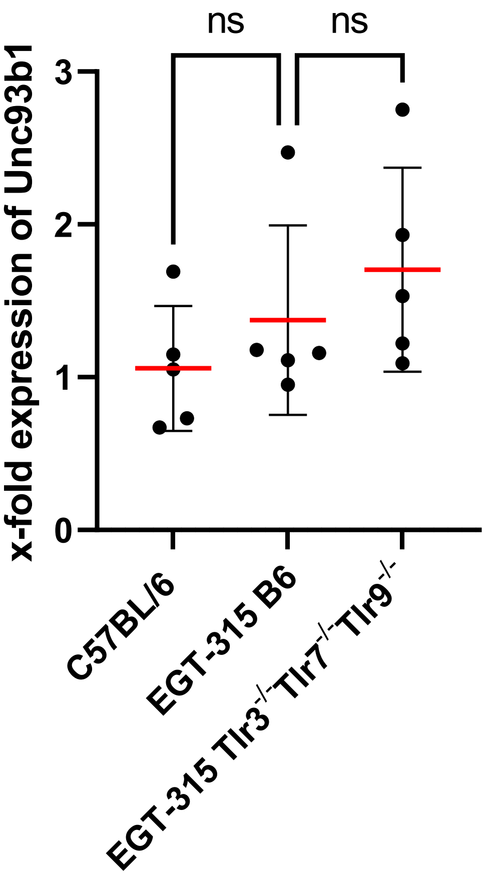
ns
ns
x-fold expression of Unc93b1
3
2
1
0
C57BL/6
EGT-315 B6
EGT-315 Tlr3-/-Tlr7-/-Tlr9-/-

h

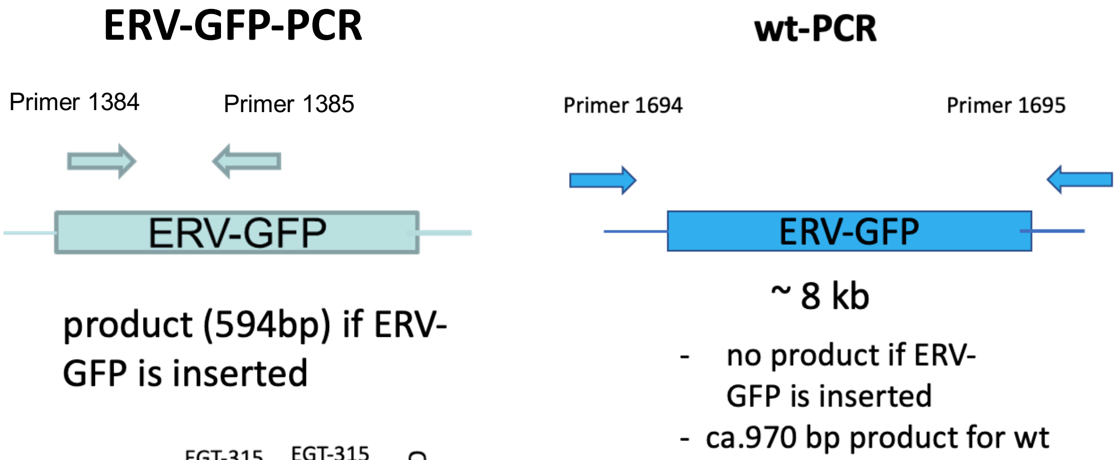
ERV-GFP-PCR
wt-PCR
Primer 1384
Primer 1385
Primer 1694
Primer 1695
ERV-GFP
ERV-GFP
~ 8 kb
product (594bp) if ERV-GFP is inserted
- no product if ERV-GFP is inserted
- ca.970 bp product for wt

i

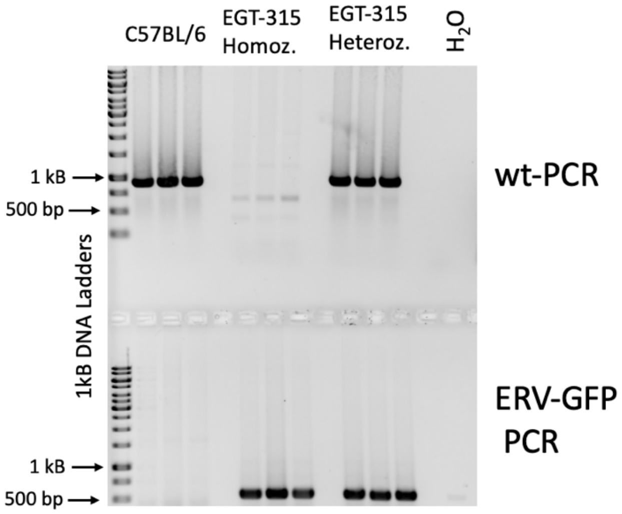
C57BL/6
EGT-315 Homoz.
EGT-315 Heteroz.
H_2O
1 kB
500 bp
1kB DNA Ladders
1 kB
500 bp
wt-PCR
ERV-GFP PCR

j

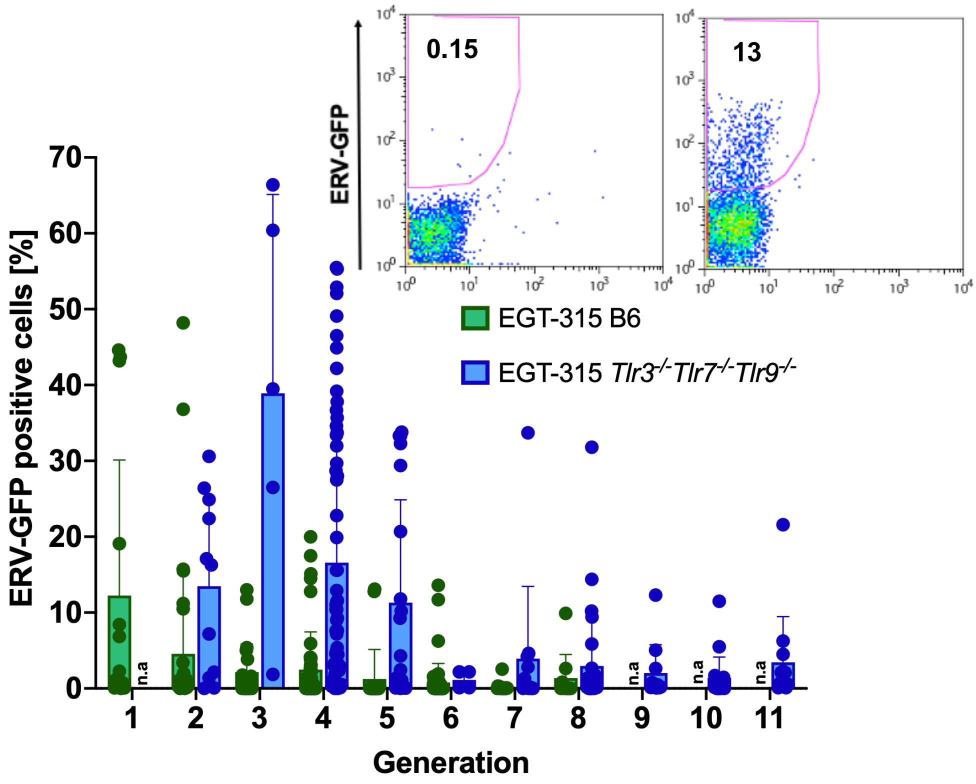
ERV-GFP
0.15
13
EGT-315 B6
EGT-315 Tlr3-/-Tlr7-/-Tlr9-/-
ERV-GFP positive cells [%]
Generation
n.a

k

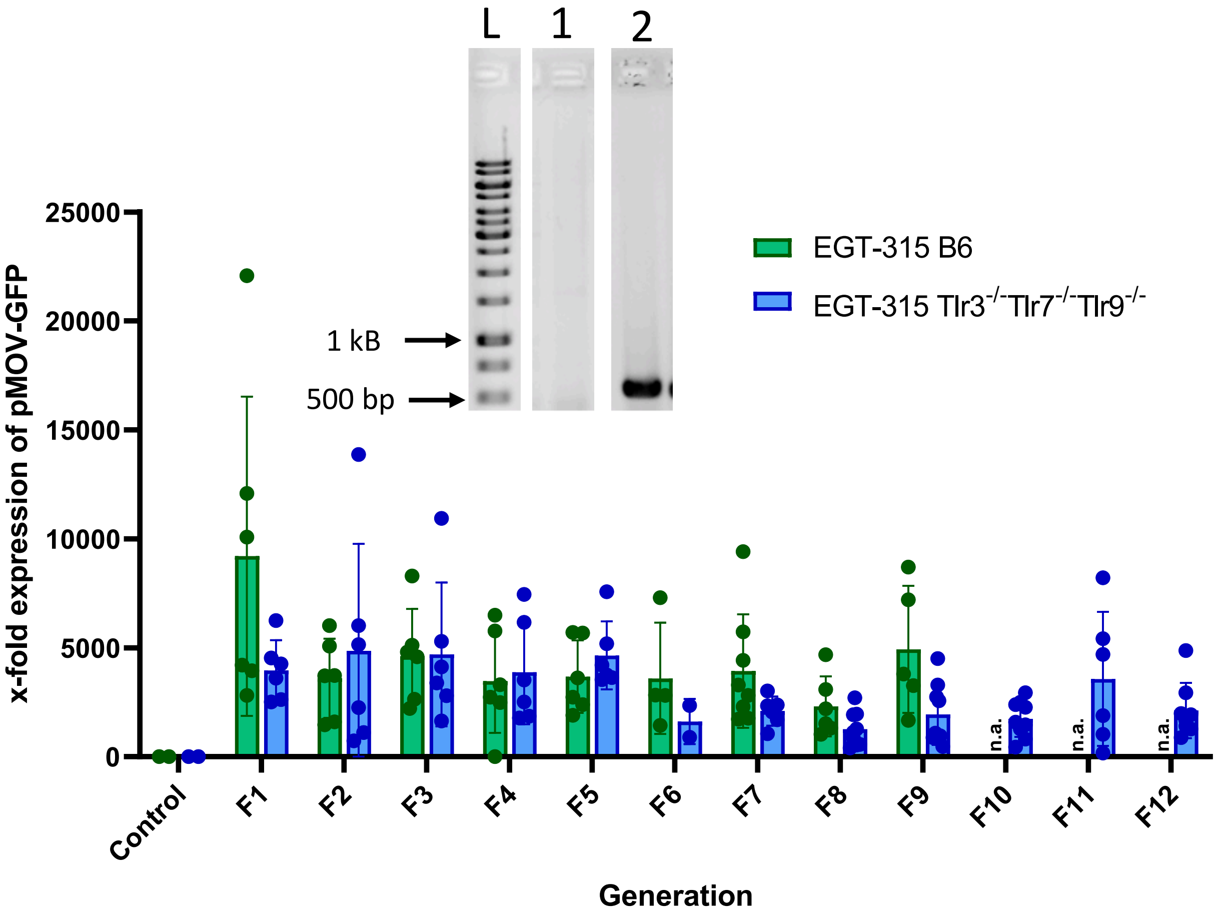

Figure S2 for Fig. 2. Genomic insertion of ERV-GFP*, Pcx expression, comparable Unc93b1 expression and detection of ERV-GFP in backcrossed EGT-315 mice*

a, **b** FISH-results on chromosomes derived of mouse from strain EGT 315 B6 with insert (a) and mouse C57BL/6 without insert (b). On the right side of (a) and (b) a metaphase is shown in inverted DAPI (4′,6-diamidino-2-phenylindole) banding; the FISH-results are shown for the same metaphases each on the right side. In (a) and (b), chromosomes 19 are labeled with whole chromosome paint (wcp, blue). Only in strain EGT 315 B6 the insert (green) could be detected in one of two chromosomes 19. **c** In the idiogram of mouse chromosome 19 is shown and an inverted DAPI-banding picture with corresponding FISH-signals for ERV-GFP (probe used Plasmid pMOV-GFP, green) in subband 19B. **d**, Whole genome sequencing of EGT-315 revealed 20 splits, reads that contain either end of pMOV-GFP (Sliva et al., 2004) (= ERV-GFP, location of GFP in Env is indicated by green bar) and the mouse genomic sequence adjacent to the insertion site. 19 overlapping splits (see Supplementary

Table 3) are located in a narrow region of 105 bps of chromosome 19 (upper part). This is the germline integration site. A single additional integration site was located in an intergenic region of chromosome 13 (lower part) and possibly represents a somatic insertion of the virus in a spleen cell(s). **e**, Genomic insertion site of ERV-GFP on Chr. 19 in the large intron of Pcx (Pyruvate carboxylase). We used the NCBI´s Blast function and the Genome Data Viewer to depict the location of the ERV-GFP insertion (red arrow). 5´-prime of the insertion site of the ERV-GFP the Lrfn4 gene (leucine rich repeat and fibronectin type III domain containing 4) is located in the Pcx intron. **f,** Pcx expression in EGT-315 B6 mice. Left, RT-QPCR using liver mRNA from individual EGT-315 B6 (EGT-315, mean= 9.43, SD= 3.65, n= 4) and C57BL/6 (B6, mean= 14.7, SD= 7.11, n= 4) mice in relative units (RU) compared to actin. Unpaired Two-tailed t test: P = 0.2392.Right, western blot of protein lysate of livers of individual mice with a anti-Pcx antibody and anti-actin Ab as control. **g**, The provirus insertion is located 630kB away from the Unc93b1 gene, a chaperon that regulates TLR function[7]. However, mRNA expression analysis does not indicate disruption of Unc93b1 expression in EGT-315 mice. Q-PCR for UNC93b1 mRNA expression of spleen cells. Actin was used to normalize expression of the specific gene. C57BL/6 (mean= 1.1, SD= 0.4, n= 5), EGT-315 B6 (mean= 1.4, SD= 0.6, n= 5), EGT-315 $Tlr3^{-/-}Tlr7^{-/-}Tlr9^{-/-}$ (mean= 1.7, SD= 0.67, n= 5), C57BL/6 vs EGT-315 B6 $^{ns}P=0.67$ and EGT-315 B6 vs EGT-315 $Tlr3^{-/-}Tlr7^{-/-}Tlr9^{-/-}$ $^{ns}P>0.20$. Statistical analysis with Tukey´s multiple comparisons test. **h**, Schematic overview of two screening PCRs spanning a region from Mo-MuLV to the GFP region of the pMOV-GFP sequence (ERV-GFP PCR, with primers 1384 and 1385) and wt-PCR, which tests for presence or absence of insertion (wt-PCR, primers 1694 and 1685). **i**, Through intercrossing of heterozygous mice we obtained viable homozygous mice at the expected frequency (Fig. S2h and S2i), which also argues against a genetic disruption of the essential Pcx gene or genes located in the vicinity of the insertion site. PCR to identify heterozygous and homozygous EGT-315 mice. Offspring of a heterozygous EGT-315 mating were tested. The PCR used primer sequences 5´ and 3´ of the ERV-GFP insertion site. A wild type allel without the insertion produces a 970 bp product while the PCR with the insertion of the ca. 8kB ERV-GFP can not produce a band due to the length of the product. **j**, ERV-GFP expression decreases in blood of EGT-315 B6 (green) and EGT-315-$Tlr3^{-/-}Tlr7^{-/-}Tlr9^{-/-}$ mice (blue) at 3 weeks of age backcrossed from (129/Sv x C57BL/6) F1 to C57BL/6. X-axis

generations of backcrosses. Each dot represents a mouse. Inserts: exemplary F10 generation of EGT-315 B6 with medium and high ERV-GFP expression (0.15% and 13%). n.a.= not available. **k**, Q-PCR for ERV-GFP presence in genomic DNA of heterozygous EGT-315 mice from different generations of EGT-315 B6 (green) and EGT-315-Tlr3$^{-/-}$Tlr7$^{-/-}$Tlr9$^{-/-}$ mice (blue). In addition all mice were tested by endpoint standard PCR using the EGT-315 endpoint standard PCR of the pMOV-GFP sequence (ERV-GFP PCR, see above Fig. S2h and i). Insert: L = 1kB DNA-Ladder, 1 = ERV-GFP negative control; 2 = EGT-315 positive mouse. Source data are provided as a Source Data file.

Supplementary Fig. S3

Rauch et al.

a

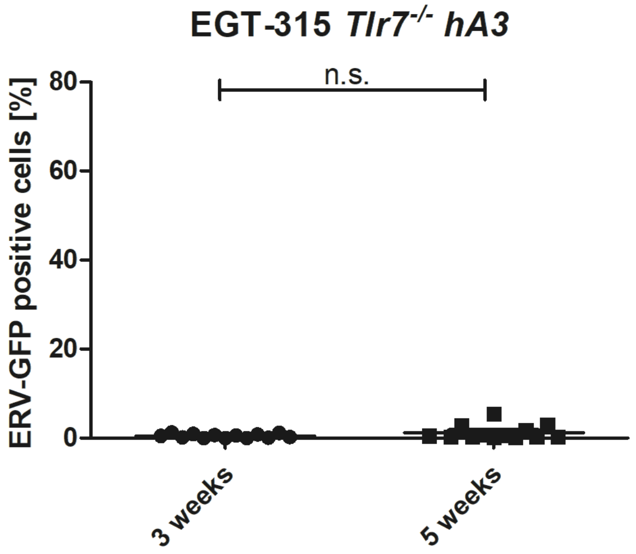

b

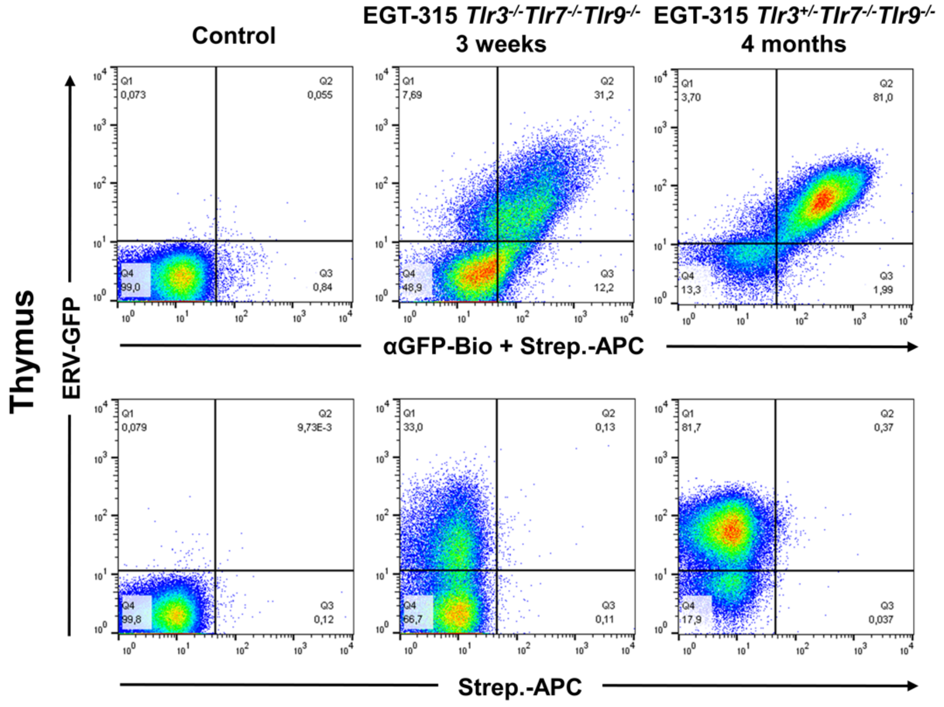

c

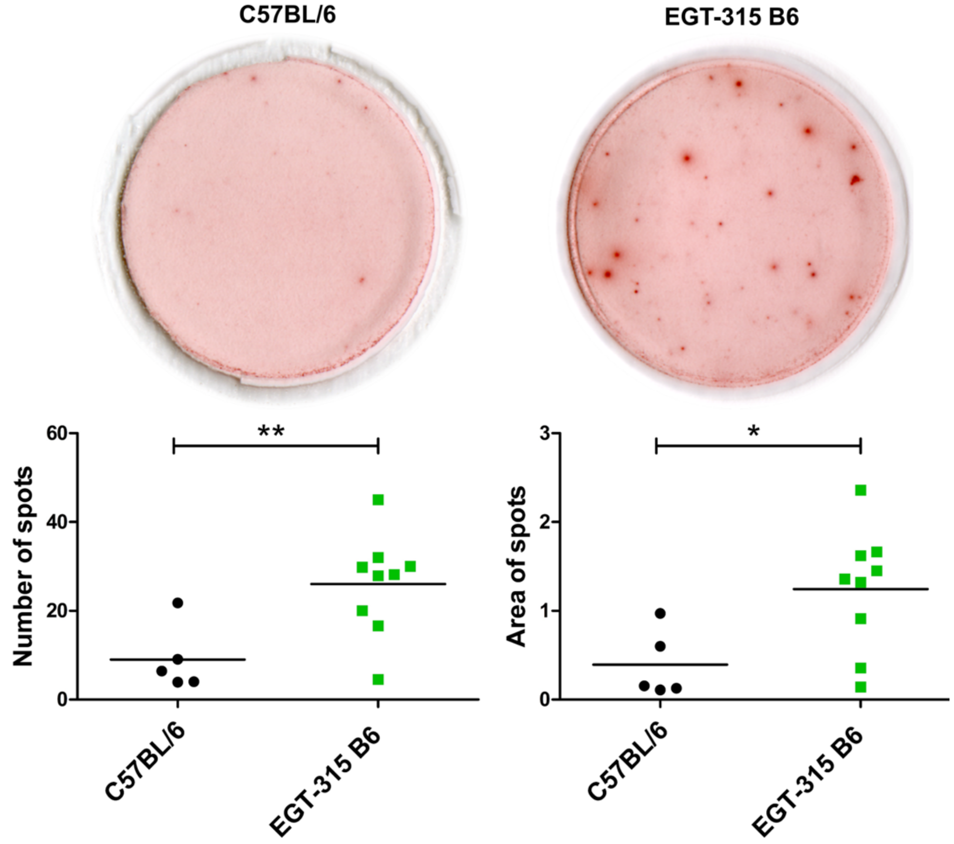
C57BL/6
EGT-315 B6
Number of spots
60
40
20
0
**
C57BL/6
EGT-315 B6
Area of spots
3
2
1
0
*
C57BL/6
EGT-315 B6

d

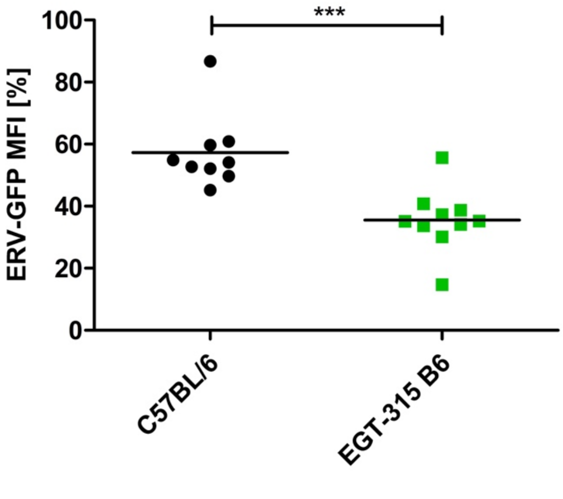
ERV-GFP MFI [%]
100
80
60
40
20
0
***
C57BL/6
EGT-315 B6

e

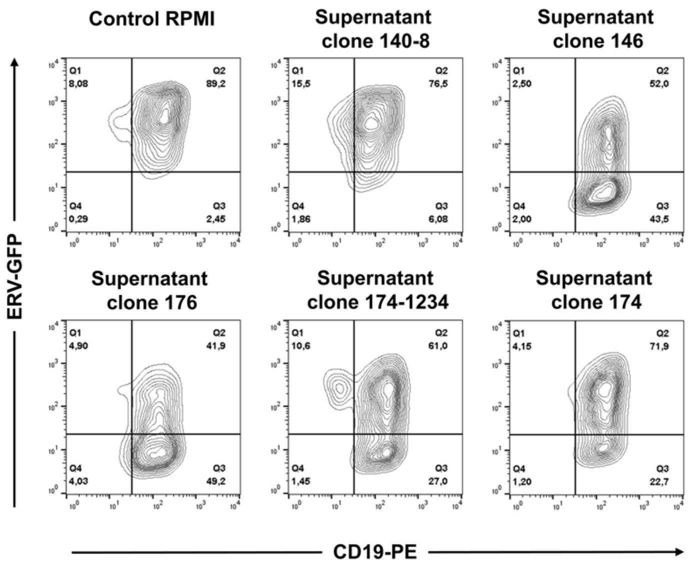

f

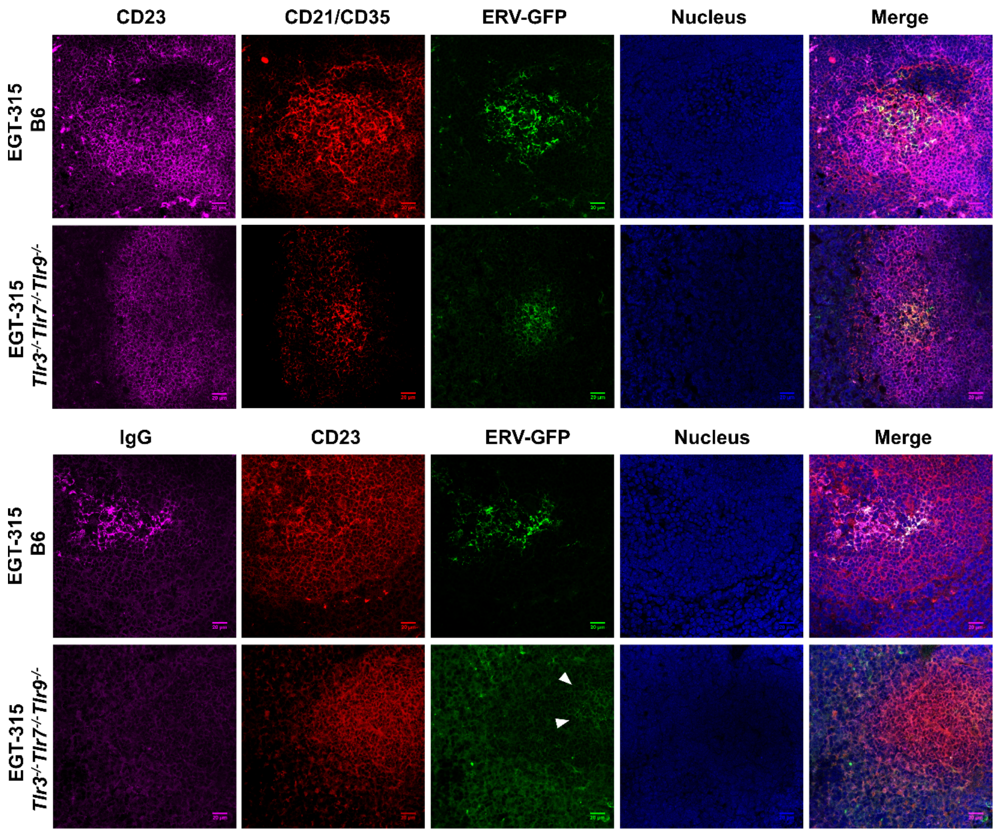

Figure S3 for Fig. 2., 3. and 4. ***Transgenic hA3 (human Apobec3) suppresses ERV-GFP expression, surface expression of Env-GFP, anti-GFP IgG producing B cells, inhibition of ERV-GFP by antibodies and spontaneous germinal centers containing ERV-GFP immune complexes on light zone FDCs***

a, Suppression of ERV-GFP expression by transgenic hA3 (human Apobec3). Flow cytometry analysis of ERV-GFP expression in peripheral blood from EGT-315 Tlr7-deficient-hA3 (transgenic hA3 mice) background (n=13) at 3 weeks and 5 weeks of age. Statistics not significant P>0.1 was done by unpaired two-tailed student´s t-test with Welch´s correction. Each dot represents a single mouse. **b**, Env-GFP expression on the surface of thymus cells of EGT-315 *Tlr3*$^{-/-}$*Tlr7*$^{-/-}$*Tlr9*$^{-/-}$ mice (3-week and 4 months old). Anti-GFP-antibody (biotinylated) plus Streptavidin-APC (upper) and Streptavidin-APC only were used (lower). **c**, ELISpot detection of B cells spontaneously expressing anti-GFP IgG antibodies. Upper part, exemplary wells from ELISpot. Lower left panel shows the mean number of spots. Right panel depicts the size of the spots as % of the wells total area. Mice analyzed were C57BL/6 control mice (black dots, n= 5) and EGT-315 B6 mice (green squares, n= 9) in 16-fold replicates. Statistics: Number of spots **P= 0.006; area of spots *P= 0.012 by unpaired two-tailed *t-test* with Welch´s correction. **d**, *In vitro* infection inhibition of ERV-GFP infection by plasma from EGT-315 B6 (green squares, n= 10) containing anti-ERV-GFP Abs and C57BL/6 controls (black dots, n= 9). Statistics used two-tailed t-test with Welch´s correction. P***= 0.007 and **e**, supernatants (1:2 dilution) of mAb from an EGT-315 B6 mouse against GFP (clones: 140-8; 146; 176; 174 and subclone 174-1234, right flow cytometry panels, n=3). WEHI-231 B cells were incubated over night with plasma or supernatant added on NIH-3T3 cells infected with ERV-GFP. Non-adherent infected WEHI-231 B cells were identified by staining with anti-CD19 and the ERV-GFP signal. ERV-GFP geometric mean fluorescent intensity (MFI). As positive control WEHI-231 B cells on NIH-3T3 ERV-GFP were incubated with RPMI-medium only. **f**, Analysis of spontaneous germinal centers. Upper panel: confocal microphotography of immune complex on the light zone FDC network (CD21/CD35) of EGT-315 B6 mice which presents ERV-GFP deposition of FDCs and is surrounded by a B cell area (CD23). Lower panel: Confocal microphotography of IgG and ERV-GFP containing immune complexes in light zone of GC of EGT-315 B6 mice but not in EGT-315 Tlr3$^{-/-}$Tlr7$^{-/-}$Tlr9$^{-/-}$. Scale bar 20 µm. Source data are provided as a Source Data file.

a

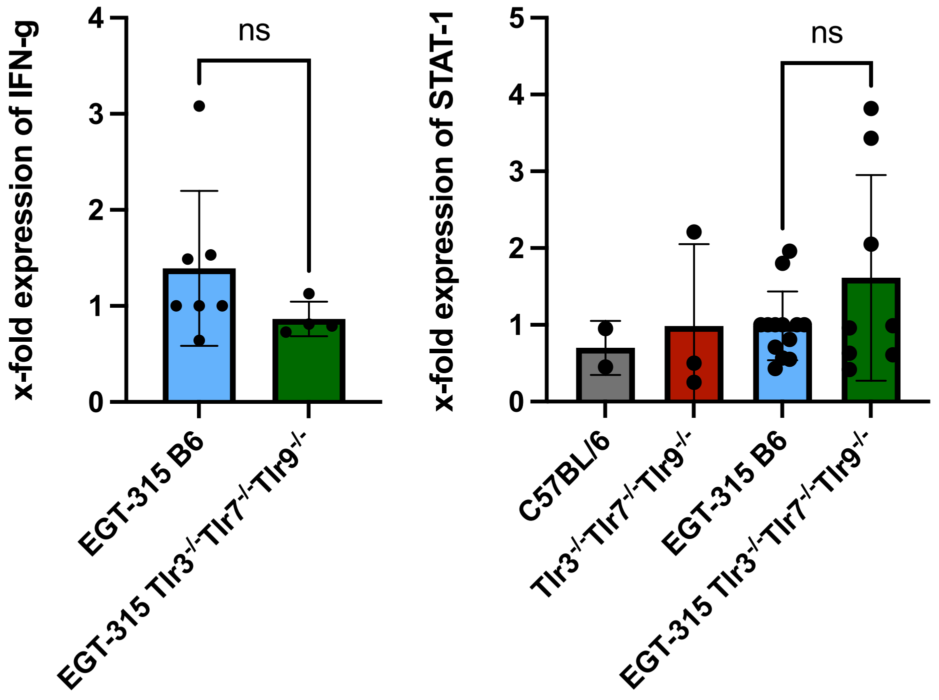
x-fold expression of IFN-g
ns
EGT-315 B6
EGT-315 Tlr3-/-Tlr7-/-Tlr9-/-
x-fold expression of STAT-1
ns
C57BL/6
Tlr3-/-Tlr7-/-Tlr9-/-
EGT-315 B6
EGT-315 Tlr3-/-Tlr7-/-Tlr9-/-

b

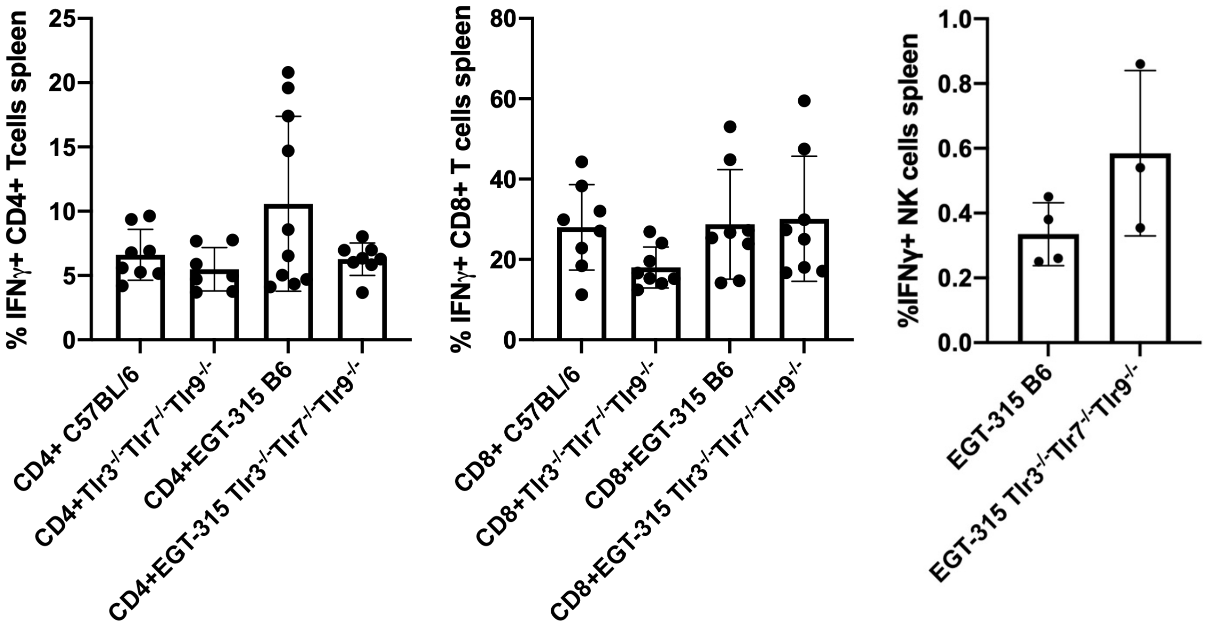
% IFNγ+ CD4+ Tcells spleen
CD4+ C57BL/6
CD4+Tlr3-/-Tlr7-/-Tlr9-/-
CD4+EGT-315 B6
CD4+EGT-315 Tlr3-/-Tlr7-/-Tlr9-/-
% IFNγ+ CD8+ T cells spleen
CD8+ C57BL/6
CD8+Tlr3-/-Tlr7-/-Tlr9-/-
CD8+EGT-315 B6
CD8+EGT-315 Tlr3-/-Tlr7-/-Tlr9-/-
%IFNγ+ NK cells spleen
EGT-315 B6
EGT-315 Tlr3-/-Tlr7-/-Tlr9-/-

c

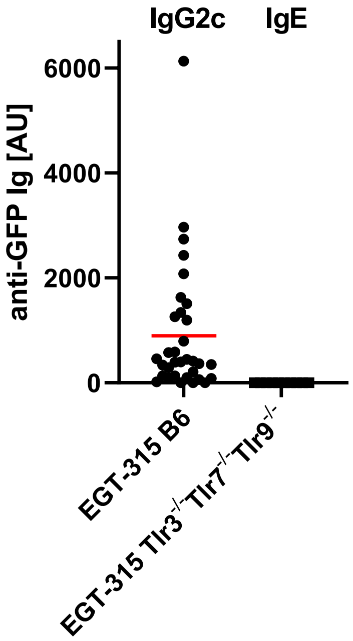

d

example for enlarged organs

example for normal organs

axillary lymph nodes

mesenteric lymph nodes

spleen

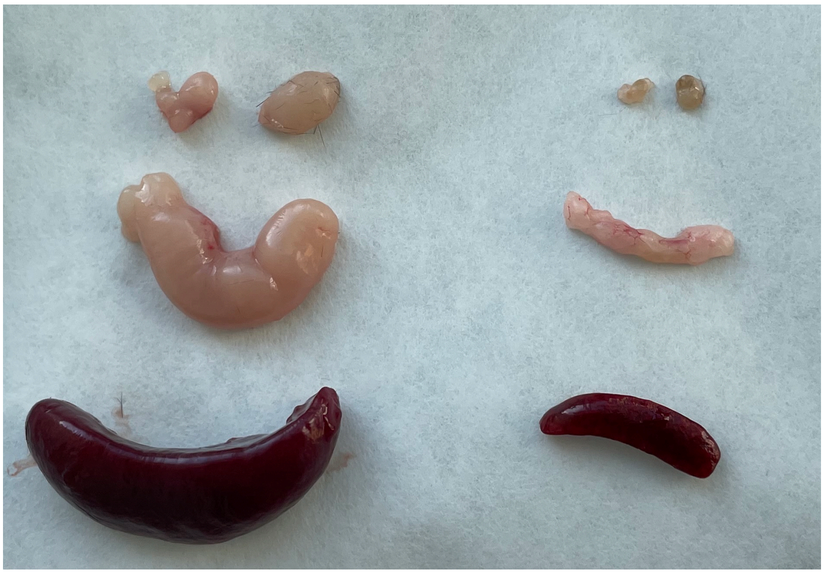

EGT-315 Tlr3$^{-/-}$Tlr7$^{-/-}$Tlr9$^{-/-}$

wt control

e

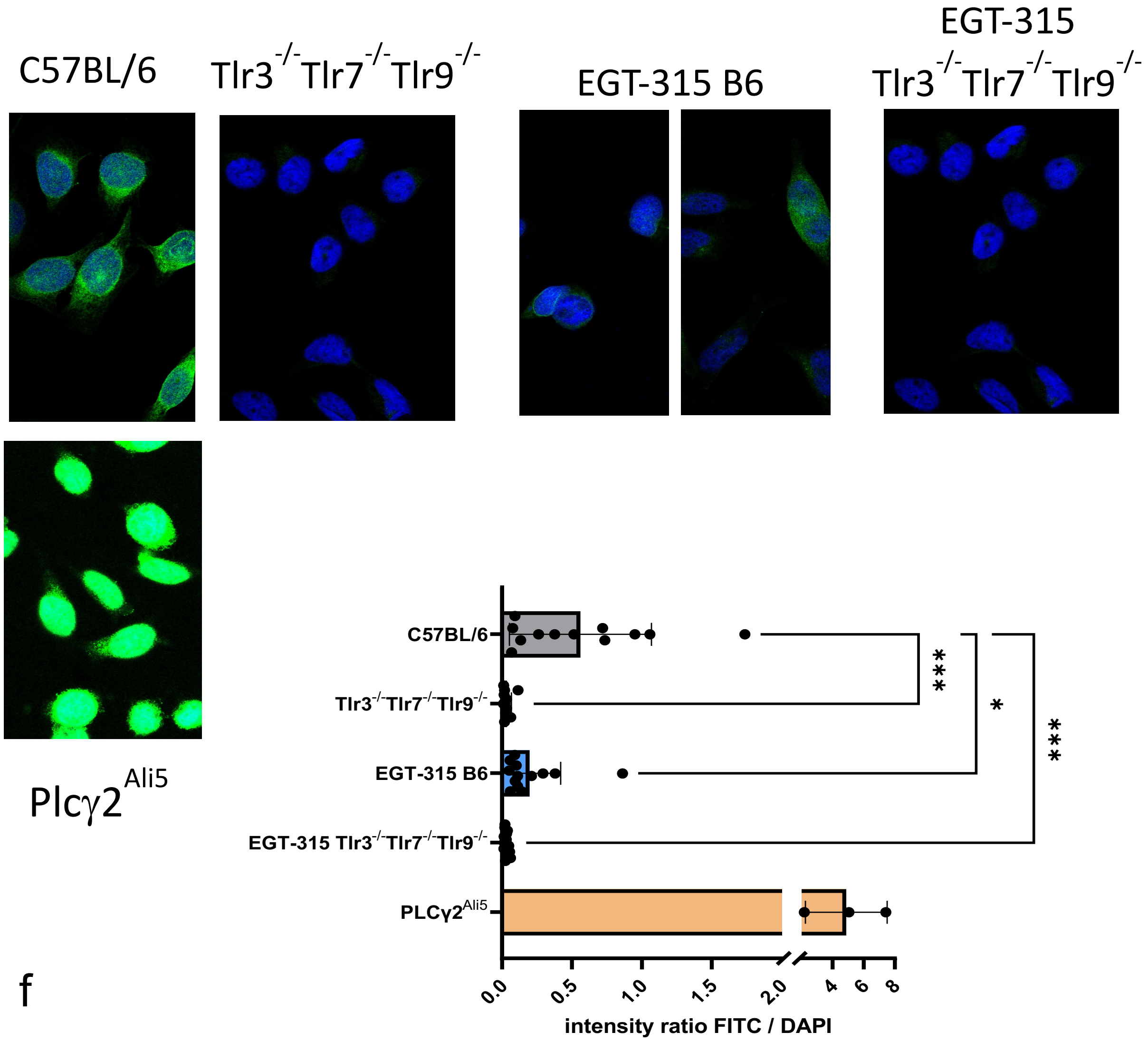

C57BL/6
Tlr3-/-Tlr7-/-Tlr9-/-
EGT-315 B6
EGT-315
Tlr3-/-Tlr7-/-Tlr9-/-
Plcγ2Ali5
C57BL/6
Tlr3-/-Tlr7-/-Tlr9-/-
EGT-315 B6
EGT-315 Tlr3-/-Tlr7-/-Tlr9-/-
PLCγ2Ali5
***
*
***
0.0
0.5
1.0
1.5
2.0
4
6
8
intensity ratio FITC / DAPI

f

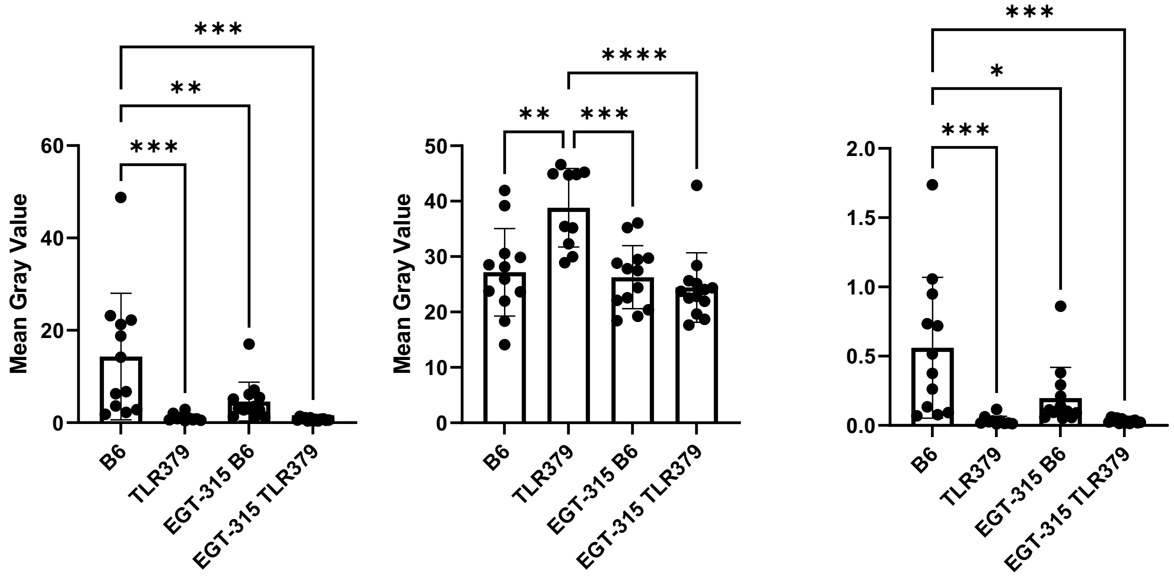

Mean Gray Value
60
40
20
0
***
**
***
B6
TLR379
EGT-315 B6
EGT-315 TLR379
Mean Gray Value
50
40
30
20
10
0
****
**
***
B6
TLR379
EGT-315 B6
EGT-315 TLR379
2.0
1.5
1.0
0.5
0.0
***
*
***
B6
TLR379
EGT-315 B6
EGT-315 TLR379

g

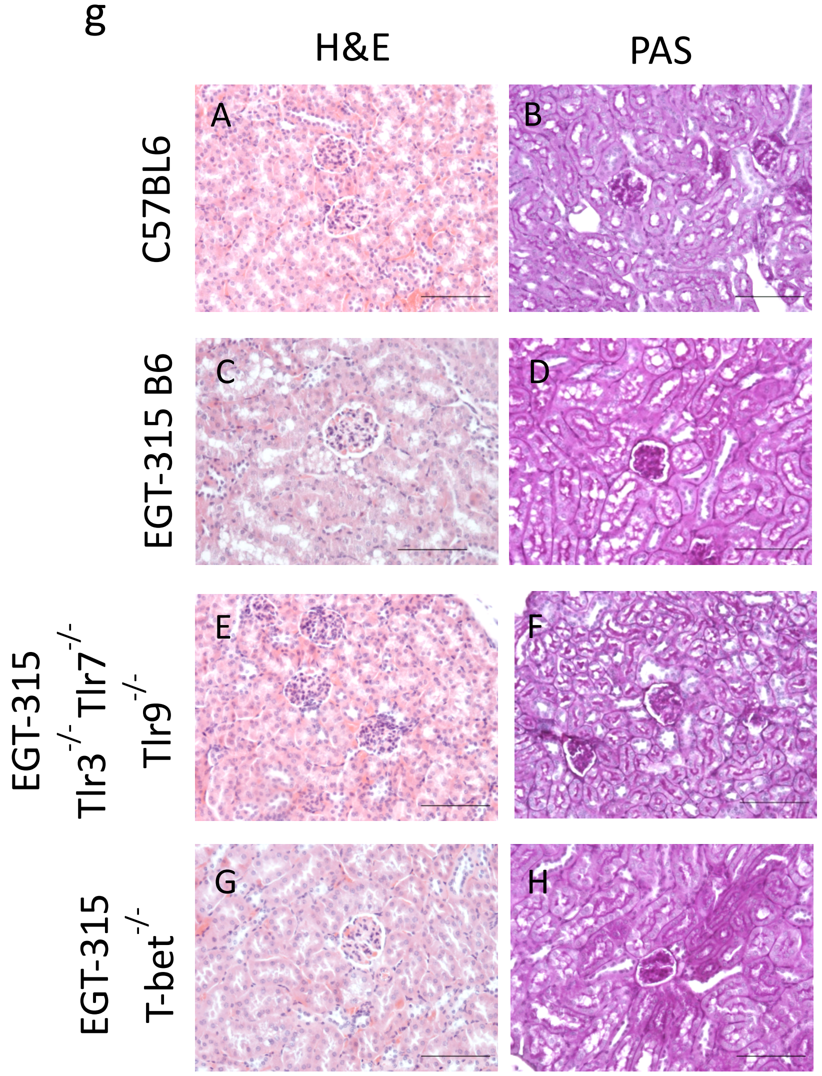

Figure S4 for Fig. 4. Q-PCR gene expressions analysis of splenic B cells and in vitro cytokine production of T- and NK cells. IgG2c and IgE anti-GFP production. T-ALL secondary lymphoid organ enlargement. HEp-2 indirect immunofluorescence assay *(IFA) and kidney histology.*

a, Q-PCR mRNA expression analysis of purified splenic B cells. Values are x-fold expression relative to houskeeping gene actin. IFN-γ (left panel) EGT-315 B6 (blue bar, mean= 1.39, SD= 0.75, n= 7), EGT-315 Tlr3$^{-/-}$Tlr7$^{-/-}$Tlr9$^{-/-}$ (green bar, mean= 0.87, SD=0.16, n= 4), Summary of 4 experiments; ^{ns}P= 0.2395. STAT-1 (right panel) C57BL/6 (black bar, mean= 0.7, SD= 0.025, n= 2), Tlr3$^{-/-}$Tlr7$^{-/-}$Tlr9$^{-/-}$ (red bar, mean= 0.99, SD= 0.87, n= 3), EGT-315 B6 (blue bar, mean= 0.99, SD= 0.43, n= 13), EGT-315 Tlr3$^{-/-}$Tlr7$^{-/-}$Tlr9$^{-/-}$ (green bar, mean= 1.61, SD= 1,2, n= 8), Summary of 7 experiments, ns is not significant, ^{ns}P= 0.1326. Statistical analysis with Tukey´s

multiple comparisons test. **b**, Intracellular cytokine flow cytometry. Purified splenic CD4^{+}T cells were stimulated with PMA/Ionomycin, incubated with Brefeldin A and then an intracellular staining for IFN-γ was performed. No significant difference could be observed in EGT-315 B6 (n=10) compared to EGT-Tlr3$^{-/-}$Tlr7$^{-/-}$Tlr9$^{-/-}$ (n= 9). Summary from 4 experiments. This result suggests that T cells are not the source of enhanced IFN-γ. Both CD8^{+} and NK cells also displayed comparable levels of IFN-γ. ***c,*** ELISA measurement of GFP-specific IgG2c in EGT-315 B6 (mean= 894, SD= 1232, n= 33) and GFP-specific IgE in EGT-315 Tlr3$^{-/-}$Tlr7$^{-/-}$Tlr9$^{-/-}$ (mean= 0, n= 10) mice. **d,** Examples of enlarged secondary lymphoid organs (lymph nodes and spleen) in mice affected by lymphoma and control mice. Related to Figure 5h. **e,** Indirect immunofluorescence (IFA) assay using HEp-2 cells. Plasma of C57BL/6 (mean= 0.56 , SD= 0.50, n=12), Tlr3$^{-/-}$Tlr7$^{-/-}$Tlr9$^{-/-}$ (mean= 0.03 , SD= 0.03; n=10), EGT-315 B6 (mean = 0.20, SD= 0.22, n=13), EGT-315 Tlr3$^{-/-}$Tlr7$^{-/-}$Tlr9$^{-/-}$ (mean = 0.03, SD= 0.02, n=13) and autoimmune Plcg2^{Ali5} (mean = 4.9, SD= 2.6, n=3), were used. An average of 20 individual cells per genotype were examined. Ratio of DAPI (nuclear staining) vs IgG-FITC is given. C57BL/6 vs Tlr3$^{-/-}$Tlr7$^{-/-}$Tlr9$^{-/-}$ ***P=0.0004. C57BL/6 vs EGT-315 B6 *P=0.011. C57BL/6 vs EGT-315 Tlr3$^{-/-}$Tlr7$^{-/-}$Tlr9$^{-/-}$ ***P=0.0001. Statistical analysis with Tukey´s multiple comparisons test. Summary of 5 experiments. **f**, For normalization of the IFA HEp-2 assay (see Fig. S4e) we counterstained the nucleus with DAPI and calculated the ratio of anti-IgG-FITC/DAPI intensities using confocal immunhistology. Signal (mean gray value) of IgG-FITC channel (left panel), DAPI (middle panel) and calculated ratio (right panel). **g**, Histopathological analysis of kidneys of different mouse strains revealed no definitive signs of glomerulonephritis. Examples of A, B: C57BL/6 (n=3); C, D: EGT-315 B6 (n=7); E, F: EGT-315 Tlr3$^{-/-}$ Tlr7$^{-/-}$ Tlr9$^{-/-}$ (n=3); G, H: EGT-315 T-bet$^{-/-}$ (n=2). Left panel (A, C, E, F): hematoxylin and eosin staining (H&E), right panel (B, D, F, H): periodic acid-Schiff. 200x magnification, scale bar: 100 µm. Source data are provided as a Source Data file.

Supplementary Fig. S5

a

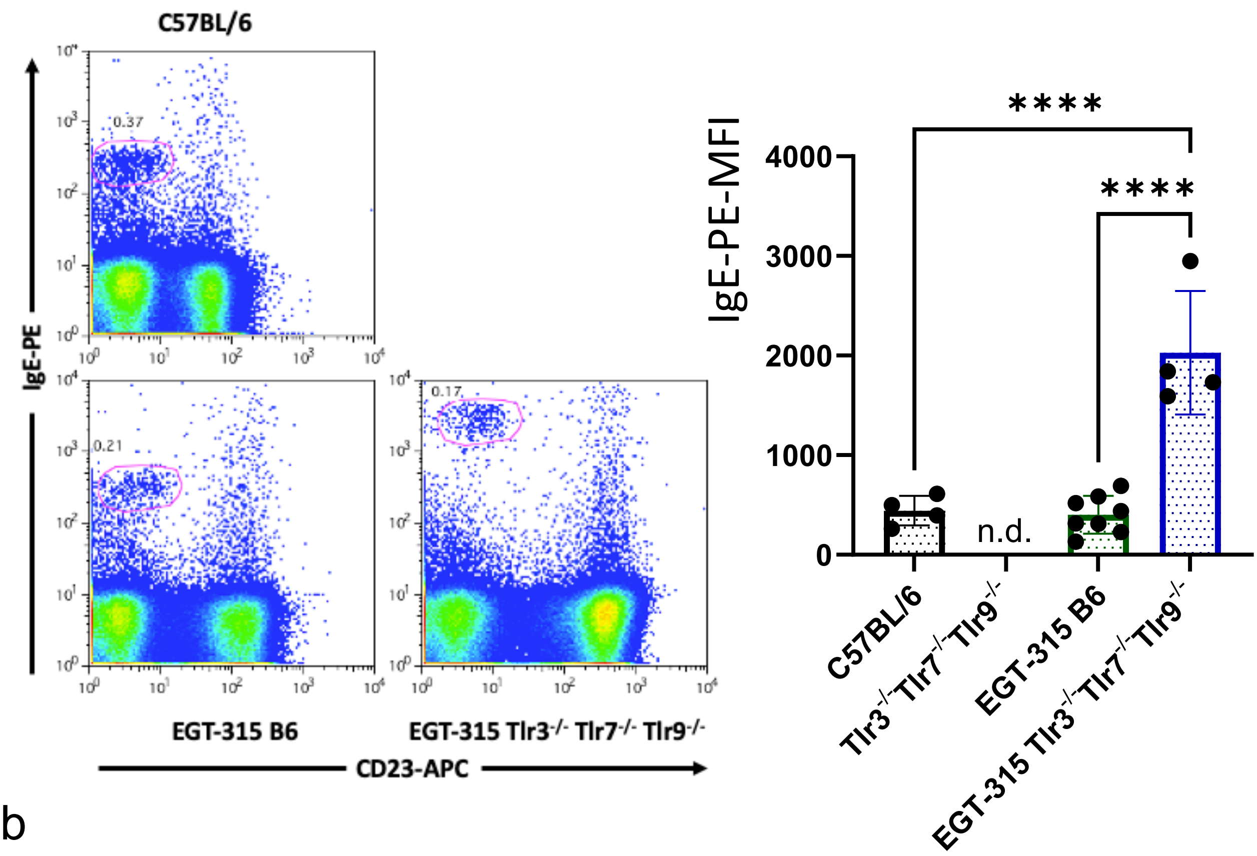

b

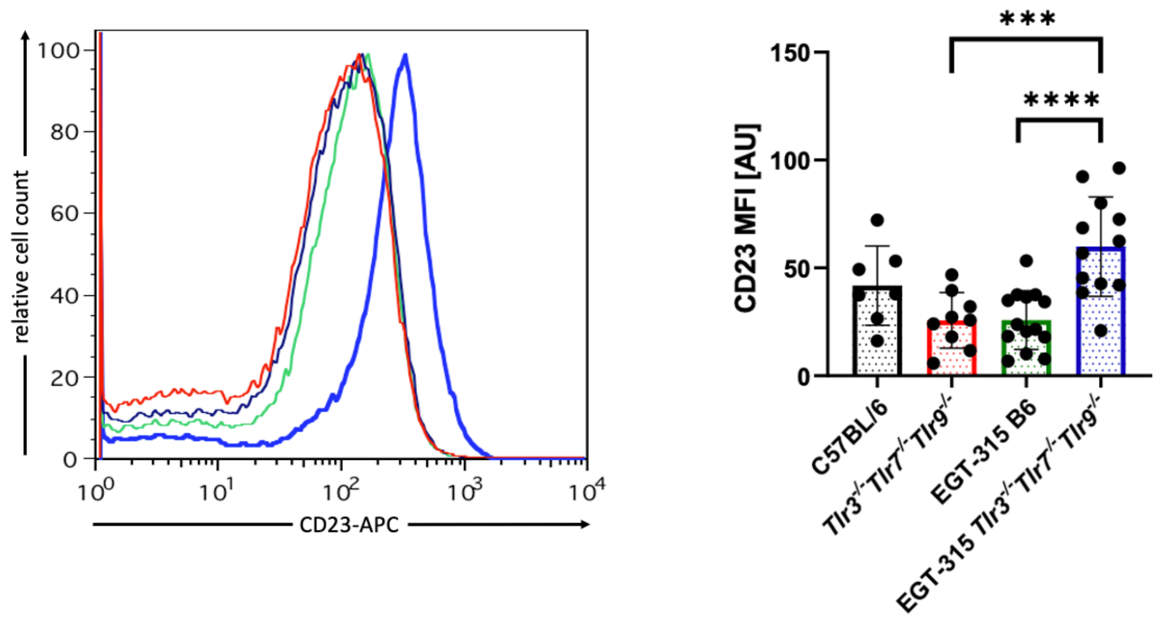

c

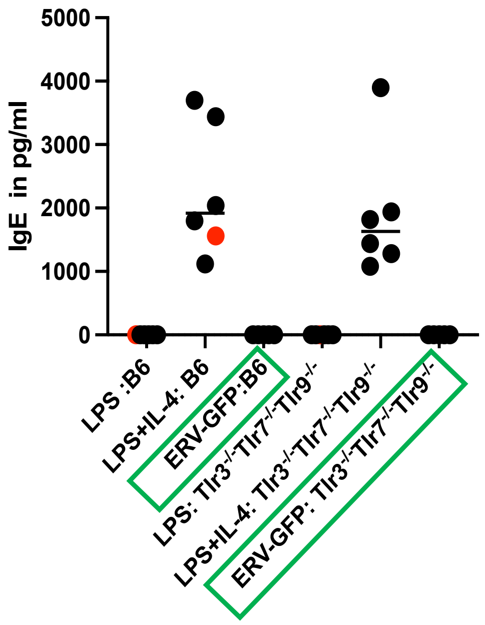

IgE in pg/ml
5000
4000
3000
2000
1000
0
LPS :B6
LPS+IL-4: B6
ERV-GFP:B6
LPS: Tlr3-/-Tlr7-/-Tlr9-/-
LPS+IL-4: Tlr3-/-Tlr7-/-Tlr9-/-
ERV-GFP: Tlr3-/-Tlr7-/-Tlr9-/-

d

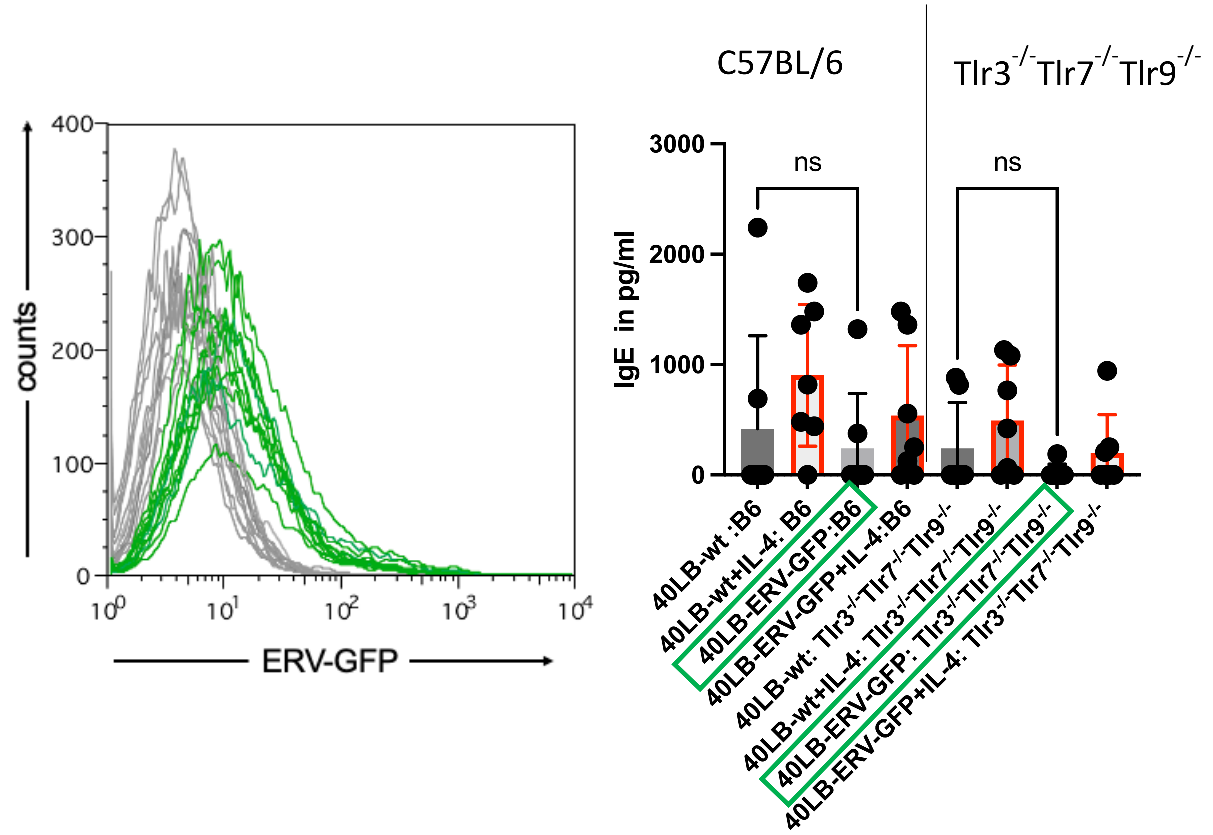

counts
400
300
200
100
0
10^0
10^1
10^2
10^3
10^4
ERV-GFP
C57BL/6
Tlr3-/-Tlr7-/-Tlr9-/-
IgE in pg/ml
3000
2000
1000
0
ns
ns
40LB-wt :B6
40LB-wt+IL-4: B6
40LB-ERV-GFP:B6
40LB-ERV-GFP+IL-4:B6
40LB-wt: Tlr3-/-Tlr7-/-Tlr9-/-
40LB-wt+IL-4: Tlr3-/-Tlr7-/-Tlr9-/-
40LB-ERV-GFP: Tlr3-/-Tlr7-/-Tlr9-/-
40LB-ERV-GFP+IL-4: Tlr3-/-Tlr7-/-Tlr9-/-

e

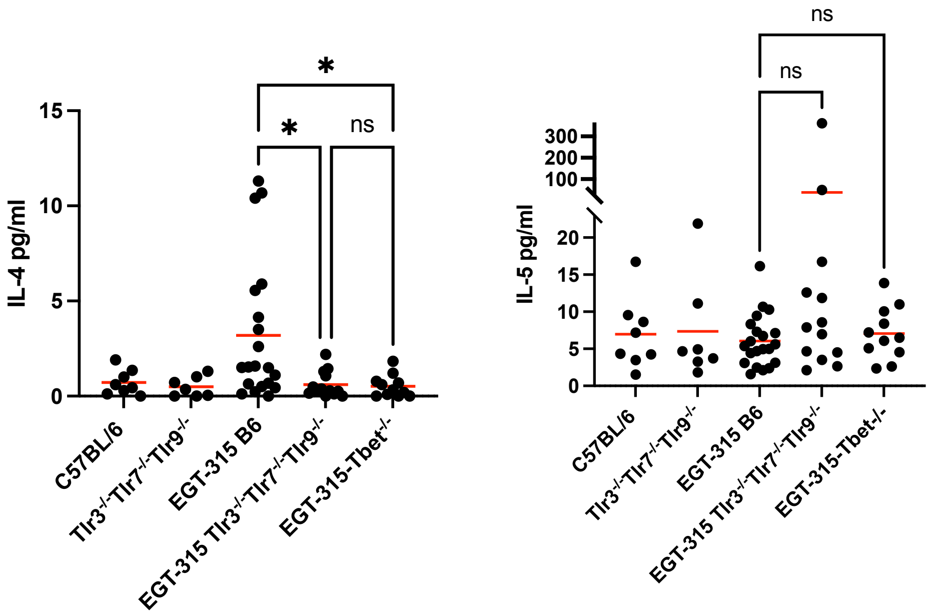
*
*
ns
IL-4 pg/ml
15
10
5
0
C57BL/6
Tlr3-/-Tlr7-/-Tlr9-/-
EGT-315 B6
EGT-315 Tlr3-/-Tlr7-/-Tlr9-/-
EGT-315-Tbet-/-
ns
ns
IL-5 pg/ml
300
200
100
20
15
10
5
0
C57BL/6
Tlr3-/-Tlr7-/-Tlr9-/-
EGT-315 B6
EGT-315 Tlr3-/-Tlr7-/-Tlr9-/-
EGT-315-Tbet-/-

f

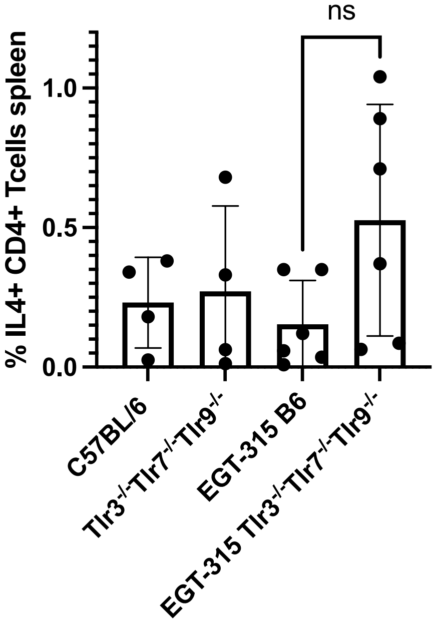
ns
% IL4+ CD4+ Tcells spleen
1.0
0.5
0.0
C57BL/6
Tlr3-/-Tlr7-/-Tlr9-/-
EGT-315 B6
EGT-315 Tlr3-/-Tlr7-/-Tlr9-/-

g

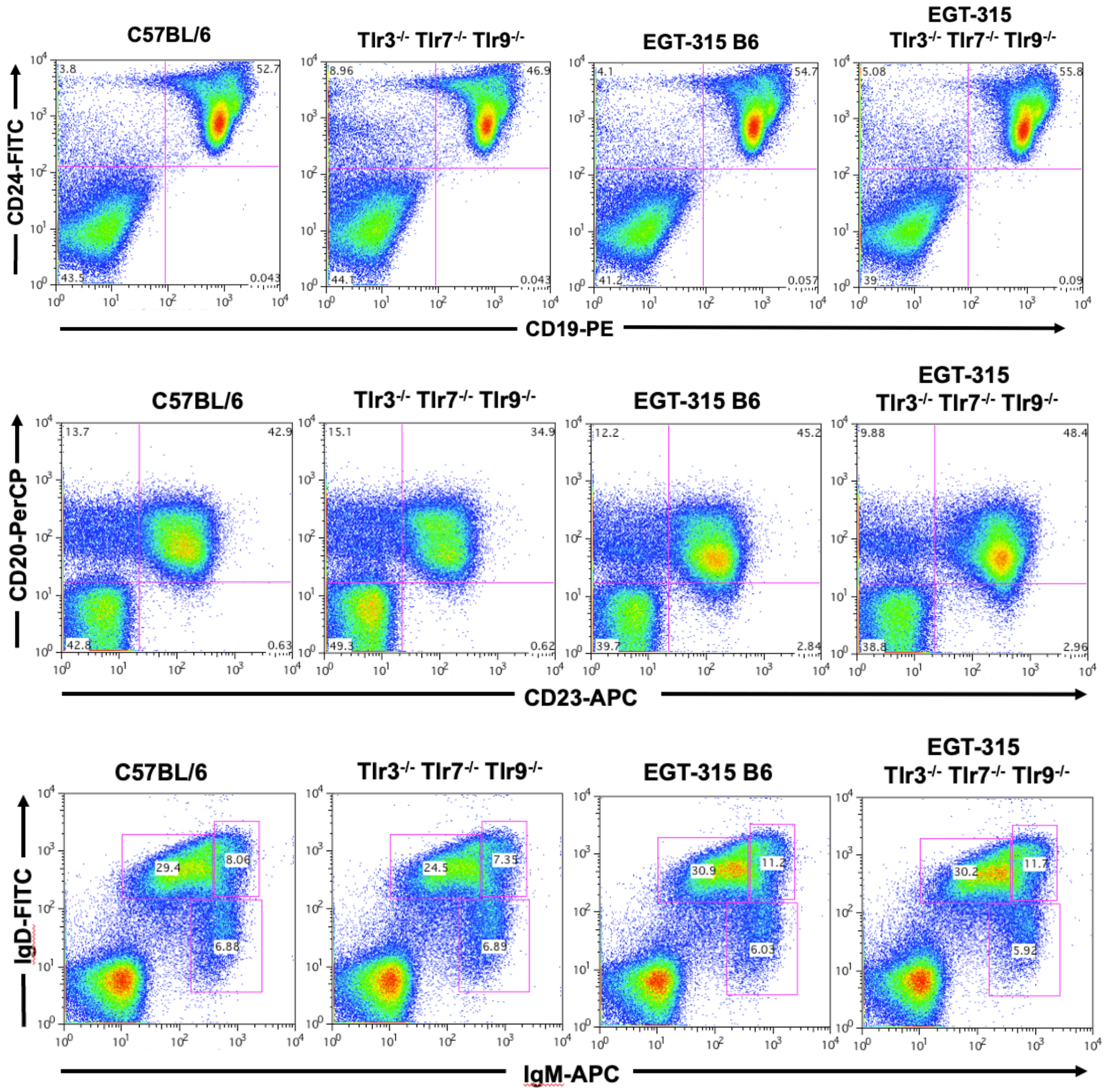
C57BL/6
Tlr3-/- Tlr7-/- Tlr9-/-
EGT-315 B6
EGT-315 Tlr3-/- Tlr7-/- Tlr9-/-
CD24-FITC
CD19-PE
3.8
52.7
43.5
0.043
8.96
46.9
44.1
0.043
4.1
54.7
41.2
0.057
5.08
55.8
39
0.09
CD20-PerCP
CD23-APC
13.7
42.9
42.8
0.63
15.1
34.9
49.3
0.62
12.2
45.2
39.7
2.84
9.88
48.4
38.8
2.96
IgD-FITC
IgM-APC
29.4
8.06
6.88
24.5
7.35
6.89
30.9
11.2
6.03
30.2
11.7
5.92

h

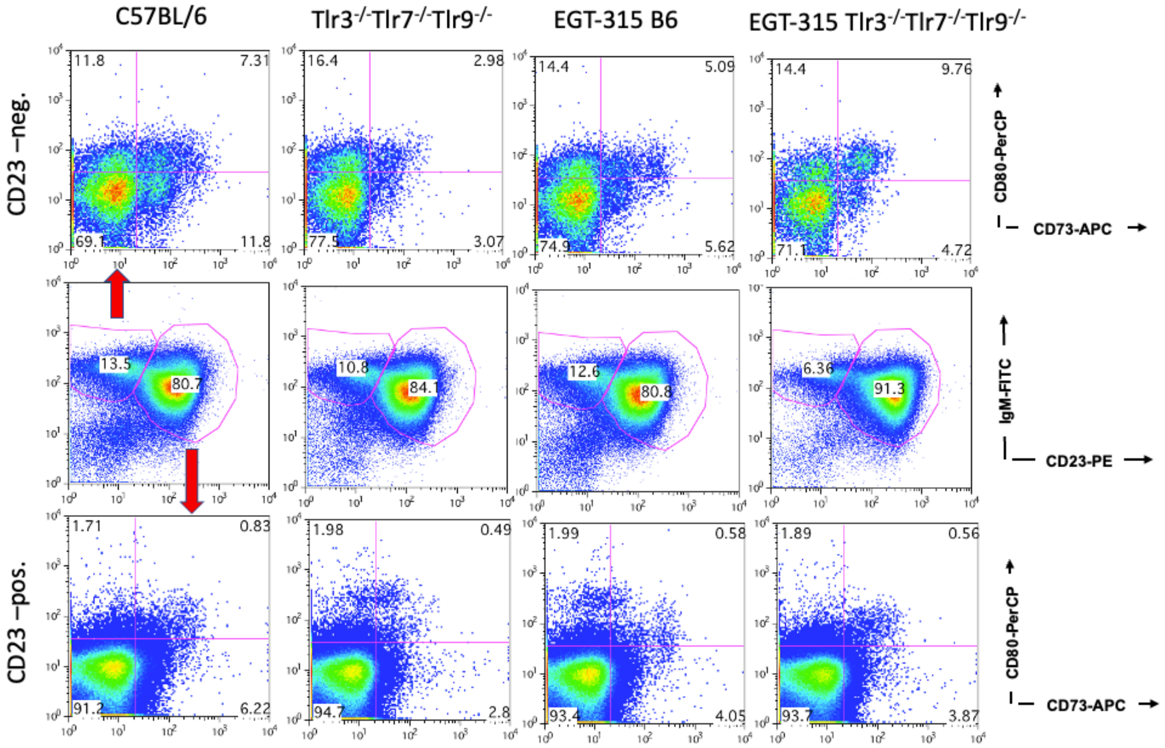
C57BL/6
Tlr3-/-Tlr7-/-Tlr9-/-
EGT-315 B6
EGT-315 Tlr3-/-Tlr7-/-Tlr9-/-
CD23 –neg.
CD23 –pos.
CD80-PerCP
CD73-APC
IgM-FITC
CD23-PE

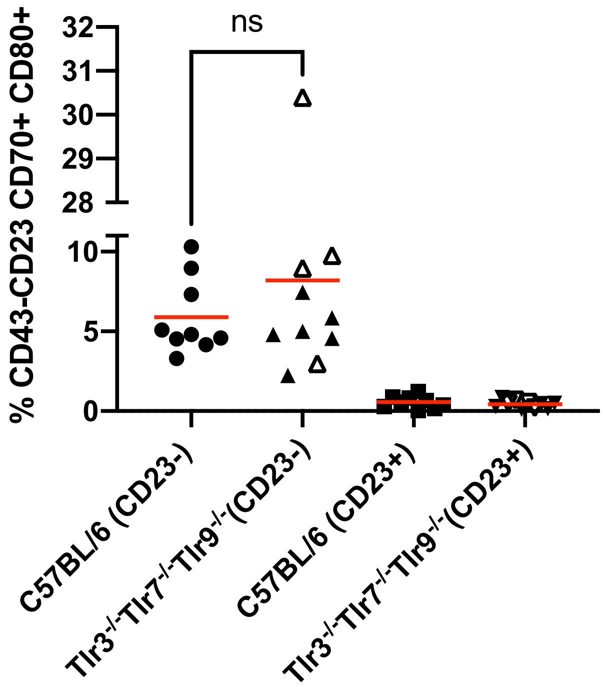
ns
% CD43-CD23 CD70+ CD80+
C57BL/6 (CD23-)
Tlr3-/-Tlr7-/-Tlr9-/-(CD23-)
C57BL/6 (CD23+)
Tlr3-/-Tlr7-/-Tlr9-/-(CD23+)

i

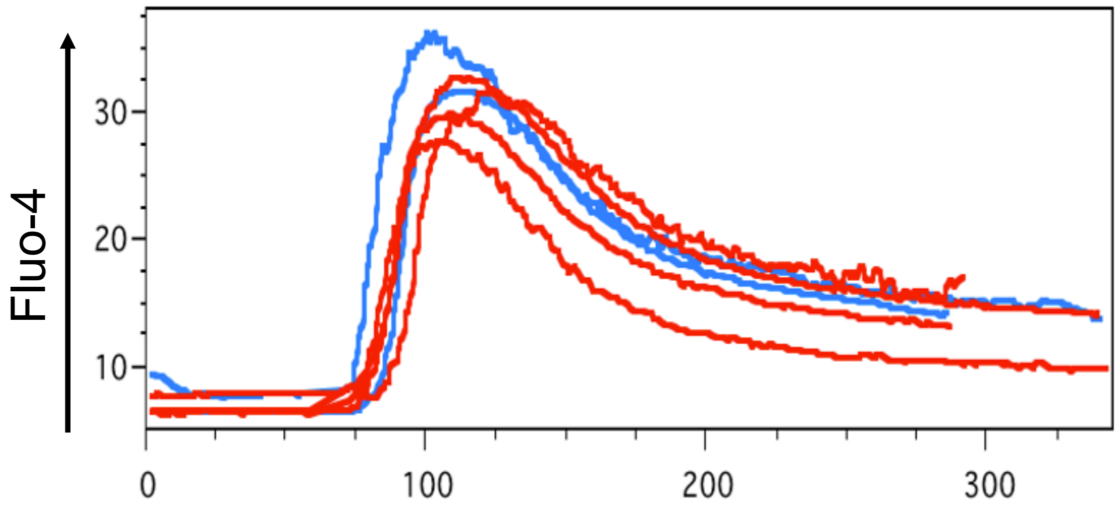
Fluo-4
30
20
10
0
100
200
300

j

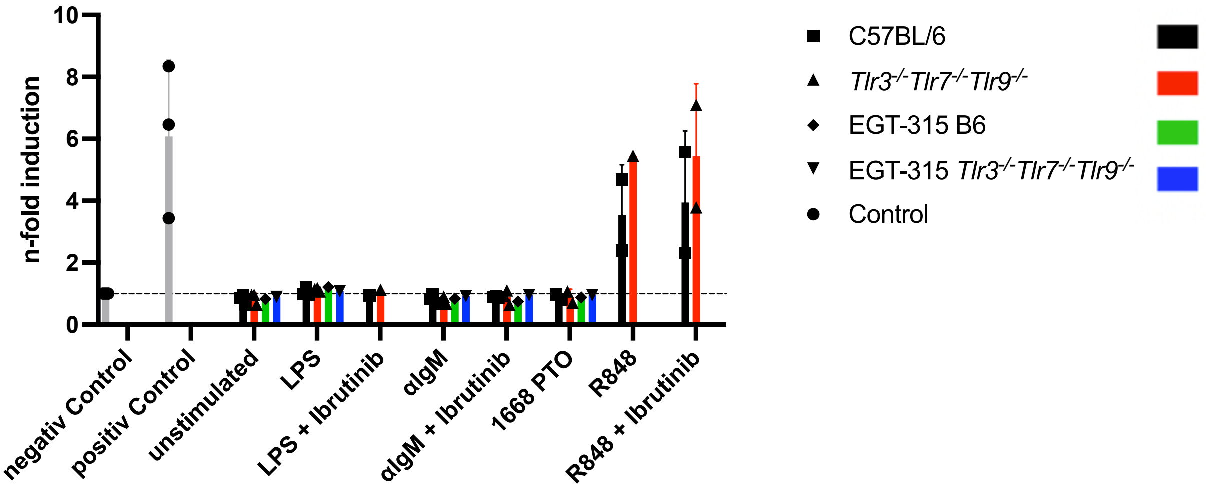
n-fold induction
10
8
6
4
2
0
negativ Control
positiv Control
unstimulated
LPS
LPS + Ibrutinib
αIgM
αIgM + Ibrutinib
1668 PTO
R848
R848 + Ibrutinib
C57BL/6
Tlr3-/-Tlr7-/-Tlr9-/-
EGT-315 B6
EGT-315 Tlr3-/-Tlr7-/-Tlr9-/-
Control

k

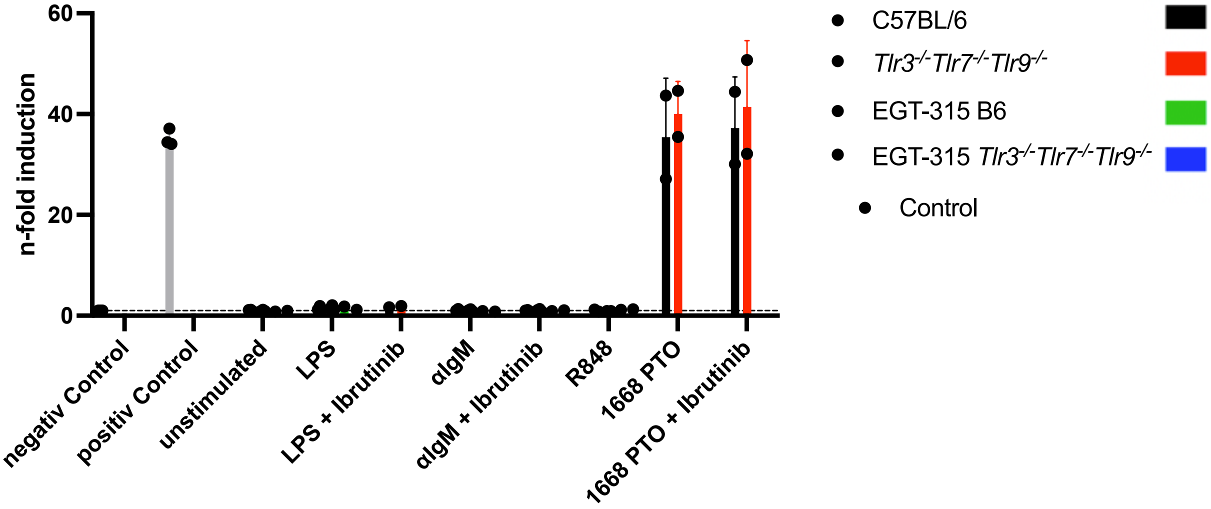
n-fold induction
60
40
20
0
negativ Control
positiv Control
unstimulated
LPS
LPS + Ibrutinib
αIgM
αIgM + Ibrutinib
R848
1668 PTO
1668 PTO + Ibrutinib
C57BL/6
Tlr3-/-Tlr7-/-Tlr9-/-
EGT-315 B6
EGT-315 Tlr3-/-Tlr7-/-Tlr9-/-
Control

Figure S5. *In vivo* binding of IgE to basophils and CD23, IgE induction in vitro, IL-4 levels in vivo and in vitro, distribution of splenic IgM$^+$ memory B cells, Ca^{2+} mobilization in EGT-315 B cells, test for Tlr7- and Tlr9 ligand release in cultured B cells. Related to Figure 6., 7. and 8.

a, Basophil-bound IgE levels measured by flow cytometry of spleen cells of C57BL/6 (black bar, mean= 441.8 , SD= 148.3, n=4), Tlr3$^{-/-}$Tlr7$^{-/-}$Tlr9$^{-/-}$ n.d. = not done, EGT-315 B6 (green bar, mean = 402.6, SD= 189.7, n=8), EGT-315 Tlr3$^{-/-}$Tlr7$^{-/-}$Tlr9$^{-/-}$ (blue bar, mean = 2028.8, SD= 620.6, n=4). Left exemplary flow cytometry panel with anti-IgE-PE and anti-CD23-APC, and right panel statistical evaluation of MFI of the IgE$^+$CD23$^-$ basophil population. C57BL/6 vs EGT-315 Tlr3$^{-/-}$Tlr7$^{-/-}$Tlr9$^{-/-}$ ****P<0.0001. EGT-315 B6 vs EGT-315 Tlr3$^{-/-}$Tlr7$^{-/-}$Tlr9$^{-/-}$ ****P<0.0001. Statistical analysis with Tukey´s multiple comparisons test. Summary of 3 experiments. **b**, CD23 expression on splenic B cells in C57BL/6 (black line and bar, mean 41.8, SD=18.4; n= 7), Tlr3$^{-/-}$Tlr7$^{-/-}$Tlr9$^{-/-}$ (red line and bar, mean= 25.7, SD= 12.9; n= 9), EGT-315 B6 (green line and bar, mean= 25.8 , SD= 13.6; n= 14) EGT-315 Tlr3$^{-/-}$Tlr7$^{-/-}$Tlr9$^{-/-}$ (blue line and bar, mean= 59.9, SD= 23.0, n= 12). Left, exemplary flow cytometry panels of splenic CD23 expression on B cells and right panel statistics of MFI. Tlr3$^{-/-}$Tlr7$^{-/-}$Tlr9$^{-/-}$ vs EGT-315 Tlr3$^{-/-}$Tlr7$^{-/-}$Tlr9$^{-/-}$ ***P=0.0004; EGT-315 B6 vs EGT-315 Tlr3$^{-/-}$Tlr7$^{-/-}$Tlr9$^{-/-}$ ****P<0.0001. Statistics with Tukey´s multiple comparisons test. Summary of 10 experiments. **c**, Splenic B cells from C57BL/6 (B6) and Tlr3$^{-/-}$Tlr7$^{-/-}$Tlr9$^{-/-}$ mice were stimulated with purified ERV-GFP virus (50ng/ml) *in vitro* for 4-5 days. As control B cells were stimulated with LPS (10μg/ml) with and w/o IL-4 (250U/ml). Only IL-4 together with LPS induced IgE production which is absent in ERV-GFP stimulated cell supernatants (green boxes). Red dot depicts single EGT-315 B6 mouse tested. **d,** IgE production of B cells from C57BL/6 or Tlr3$^{-/-}$Tlr7$^{-/-}$Tlr9$^{-/-}$ mice cocultured with 40LB wild type and 40LB-ERV-GFP (40LB expressing ERV-GFP). Left panel, B cells were harvested and stained with anti-CD45RB-B220 and gated for B cells. Green lines show B cells from individual cocultures with either CD40LB-wt (grey lines) or positive for ERF-GFP 72h after start of co-culture (green lines). Right panel, Supernatant was taken after 4-5 days. In both genotypes presence of ERV-GFP$^+$ 40LB cells (green boxes) did not induced IgE production. Addition of IL-4 as positive control induced IgE secretion in this system (red bars). 40LB-wt:B6 (mean= 419,SD= 843, n=7), 40LB-wt+IL-4:B6 (mean= 903,SD= 640, n=7), 40LB-ERV-GFP:B6 (mean= 242 ,SD= 496, n=7), 40LB-ERV-GFP+IL-4:B6 (mean= 539,SD= 632, n=7), 40LB-wt:Tlr3$^{-/-}$Tlr7$^{-/-}$Tlr9$^{-/-}$ (mean= 242 ,SD= 414, n=7), 40LB-wt+IL-4: Tlr3$^{-/-}$Tlr7$^{-/-}$Tlr9$^{-/-}$(mean= 494 ,SD= 501, n=7), 40LB-ERV-GFP: Tlr3$^{-/-}$Tlr7$^{-/-}$Tlr9$^{-/-}$(mean=27 ,SD=71.4,

n=7), 40LB-ERV-GFP+IL-4: Tlr3$^{-/-}$Tlr7$^{-/-}$Tlr9$^{-/-}$ (mean= 201 ,SD= 346, n=7). 40LB-wt:B6 vs 40LB-ERV-GFP:B6 ^{ns}P= 0.9985. 40LB-wt:Tlr3$^{-/-}$Tlr7$^{-/-}$Tlr9$^{-/-}$ vs 40LB-ERV-GFP: Tlr3$^{-/-}$Tlr7$^{-/-}$Tlr9$^{-/-}$ ^{ns}P= 0.9687. Tukey´s multiple comparisons test. Summary of 3 experiments. **e,** Serum cytokine bead array (Becton Dickinson) of serum probes of indicated mice. Left, IL-4 measurement of serum samples from C57BL/6 (mean= 0.718, SD= 0.659, n= 8), Tlr3$^{-/-}$Tlr7$^{-/-}$Tlr9$^{-/-}$(mean= 0.494, SD= 0.529, n=7), EGT-315 B6 (mean= 3.20, SD= 3.70, n= 20), EGT-315 Tlr3$^{-/-}$Tlr7$^{-/-}$Tlr9$^{-/-}$ (mean= 0.612, SD= 0.677, n=13) and EGT-315 T-bet$^{-/-}$ (mean= 0.522, SD= 0.585, n=11) were tested with BD cytokine bead array using a flowcytometer. EGT-315 B6 vs EGT-315 Tlr3$^{-/-}$Tlr7$^{-/-}$Tlr9$^{-/-}$ *P= 0.0175. EGT-315 B6 vs EGT-315 T-bet$^{-/-}$ *P= 0.0205. EGT-315 Tlr3$^{-/-}$Tlr7$^{-/-}$Tlr9$^{-/-}$ vs EGT-315 T-bet$^{-/-}$ ^{ns}P= >0.9999. Tukey´s multiple comparisons test. Summary of 3 experiments. Right, IL-5 measurement of Serum samples from C57BL/6 (mean= 6.96, SD= 4.80, n= 8), Tlr3$^{-/-}$Tlr7$^{-/-}$Tlr9$^{-/-}$(mean= 7.34, SD= 7.06, n=7), EGT-315 B6 (mean= 6.04, SD= 3.52, n= 21) EGT-315 Tlr3$^{-/-}$Tlr7$^{-/-}$Tlr9$^{-/-}$ (mean= 37.9, SD= 98.0, n=13). EGT-315 T-bet$^{-/-}$(mean= 7.06, SD= 3.55, n=11) were tested with BD cytokine bead array using a flowcytometer. EGT-315 B6 vs EGT-315 Tlr3$^{-/-}$Tlr7$^{-/-}$Tlr9$^{-/-}$ ^{ns}P=0.2954. EGT-315 B6 vs EGT-315 T-bet$^{-/-}$ ^{ns}P= >0.9999. Tukey´s multiple comparisons test. Summary of 3 experiments. **f,** Intracellular cytokine flow cytometry. Purified splenic CD4^{+}T cells were stimulated with PMA/Ionomycin, incubated with Brefeldin A and then an intracellular staining for IL-4 was performed. No significant difference could be observed in EGT-315 B6 (n=6) compared to EGT-Tlr3$^{-/-}$Tlr7$^{-/-}$Tlr9$^{-/-}$ (n= 6). Summary from 3 experiments. This result suggests that T cells are not the only source of IL-4 in EGT-Tlr3$^{-/-}$Tlr7$^{-/-}$Tlr9$^{-/-}$ which could contribute to enhanced IgE levels. 2 way ANOVA, Tukey´s multiple comparisons test. **g**, Exemplary flowcytometry of spleen cells from C57BL/6 (n= 3), Tlr3$^{-/-}$Tlr7$^{-/-}$Tlr9$^{-/-}$ (n= 2), EGT-315 B6 (n= 6), EGT-315 Tlr3$^{-/-}$Tlr7$^{-/-}$Tlr9$^{-/-}$ (n= 5) using the B cell markers CD24 with CD19; CD20 with CD23; IgD with IgM. Summary of 3 experiments. **h**, IgM^{+} memory B cells in Tlr3$^{-/-}$Tlr7$^{-/-}$Tlr9$^{-/-}$ mice. Splenic B cells negative selected by CD43 MACS were gated for either CD43^{-}IgM^{medium}CD23^{+} or CD43^{-}IgM^{high}CD23^{-} B cells and stained for CD80 and CD73 (IgM^{+} memory B cells). Age of mice 7-24 weeks. CD80/CD73 double positive IgM^{+} B cells. CD43^{-}IgM^{medium}CD23^{+}: C57BL/6 and EGT-315 B6 (squares, mean= 0.57, SD= 0.38, n= 9), Tlr3$^{-/-}$Tlr7$^{-/-}$Tlr9$^{-/-}$ and EGT-315 Tlr3$^{-/-}$Tlr7$^{-/-}$Tlr9$^{-/-}$ (triangles downward, mean= 0.43, SD= 0.24 , n= 10). CD43^{-}IgM^{high}CD23^{-}: C57BL/6 and EGT-315 B6 (dots, mean= 5.89, SD= 2.4, n= 9), Tlr3$^{-/-}$Tlr7$^{-/-}$Tlr9$^{-/-}$ and EGT-315 Tlr3$^{-/-}$Tlr7$^{-/-}$Tlr9$^{-/-}$ (triangles upward, mean= 8.20, SD= 8.16 , n= 10), ^{ns}P=0.66.

Summary of 5 experiments, 2 way ANOVA, Tukey´s multiple comparisons test. **i**, Measurement of Ca^{2+}-mobilization induced by anti-IgM. Purified B cells from C57BL/6 (blue, n= 2) and Tlr3$^{-/-}$Tlr7$^{-/-}$Tlr9$^{-/-}$ (red, n= 4) were loaded with the Ca^{2+}-indicator dye Fluo-4. Cells were stimulated with anti-IgM and kinetics of cytoplasmatic Ca^{2+} were measured. **j**, Test for presence of TLR7 ligands in *in vitro* culture. HEK-Blue hTlr7 cells were stimulated with supernatant of stimulated B cells C57BL/6 (black bars, n= 3), *Tlr3$^{-/-}$Tlr7$^{-/-}$Tlr9$^{-/-}$* (red bars, n= 3), EGT-315 B6 (green bar, n= 1) and EGT-315 *Tlr3$^{-/-}$Tlr7$^{-/-}$Tlr9$^{-/-}$* (blue bar, n= 1), medium as negative control and R848 as positive control (grey bars) and fold induction was determined after 18 hours, comparing unstimulated vs stimulated reporter gene expression. Experiments were repeated 3 times. **k**, Test for presence of TLR9 ligands in *in vitro* culture. HEK293 mTlr9 reporter cells were stimulated with supernatant of stimulated B cells (C57BL/6 (black bars, n= 3), *Tlr3$^{-/-}$Tlr7$^{-/-}$Tlr9$^{-/-}$* (red bars, n=3), EGT-315 B6 (green bar, n= 1) and EGT-315 *Tlr3$^{-/-}$Tlr7$^{-/-}$Tlr9$^{-/-}$* (blue bar, n= 1), medium as negative control and 1668 PTO ODN as positive control (grey bars) and fold induction was determined after 18 hours, comparing unstimulated vs stimulated reporter gene expression. Experiments were repeated 3 times. In every cell culture a number of cells die and disintegrate. This leads to release of both DNA and RNA of eukaryotic origin. To test if these potential Tlr7- and Tlr9-ligands might play a role in providing an additional signal to the BCR signal through anti-IgM we used reporter cell lines which are able to respond to Tlr7 and Tlr9 ligands. We used reporter HEK cell lines which contain either hTLR7 and the QuantiBlue reporter system or mTlr9 coupled to luciferase reporter system. Addition of *bona fide* R848-Tlr7- or 1668-Tlr9 ligands induced 7-fold and 37-fold reporter induction respectively. However, none of the supernatants of B cells cultured for 3 days with different stimuli did activate the reporter coupled to either Tlr7 or Tlr9. Therefore we concluded that absence of anti-IgM driven B cell proliferation/survival is not due to lack of sensing of eukaryotic RNA/DNA during the culture of *Tlr3$^{-/-}$Tlr7$^{-/-}$Tlr9$^{-/-}$* B cells but must reflect a direct impaired BCR signal caused by the absence of Tlrs. Source data are provided as a Source Data file.

Supplementary Fig. S6

Rauch et al.

a

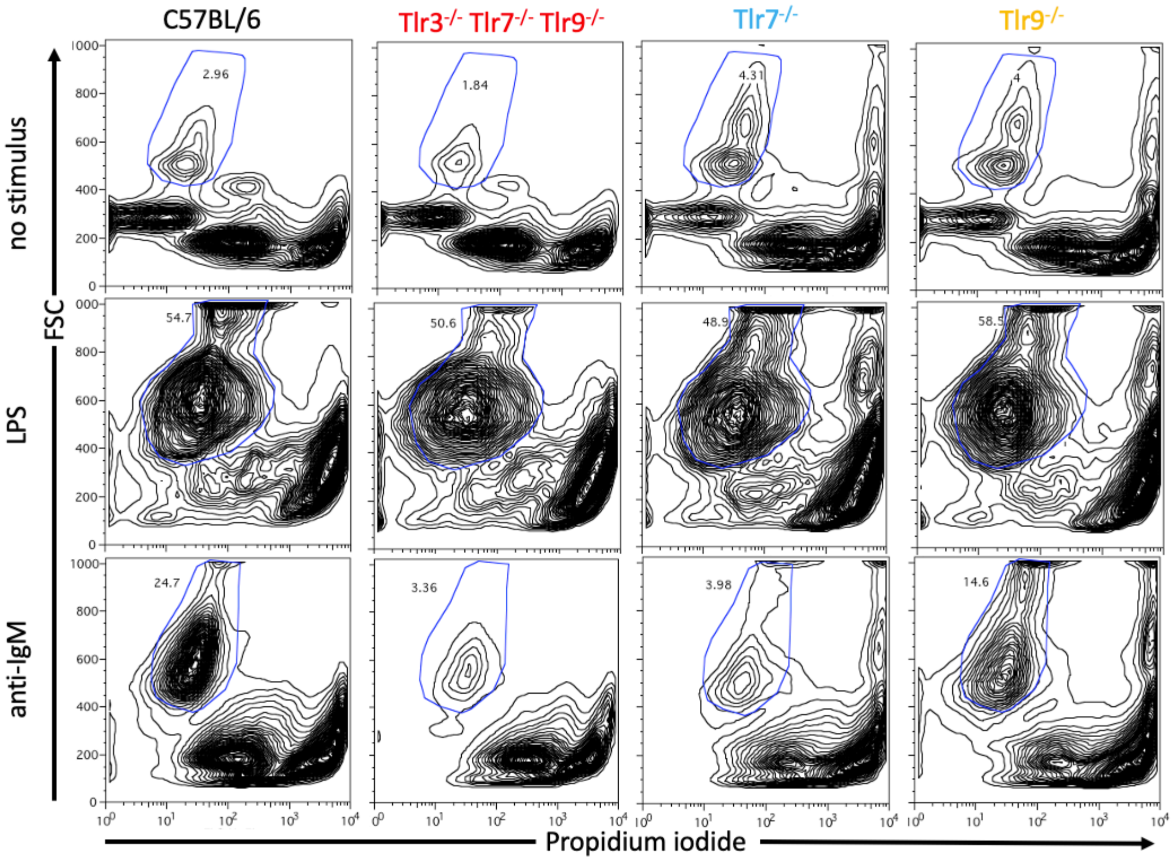

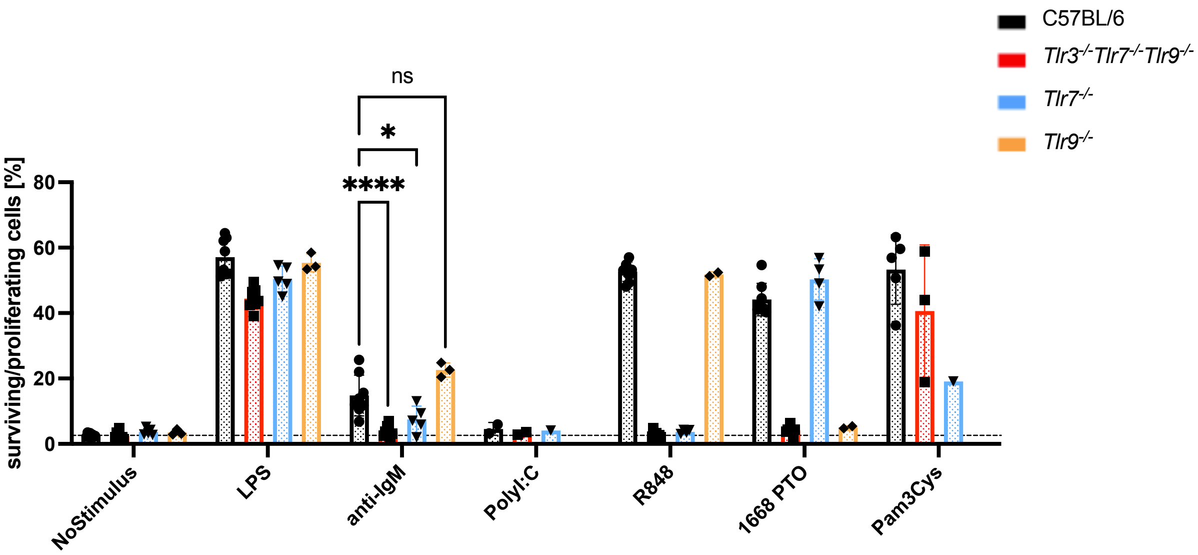

b

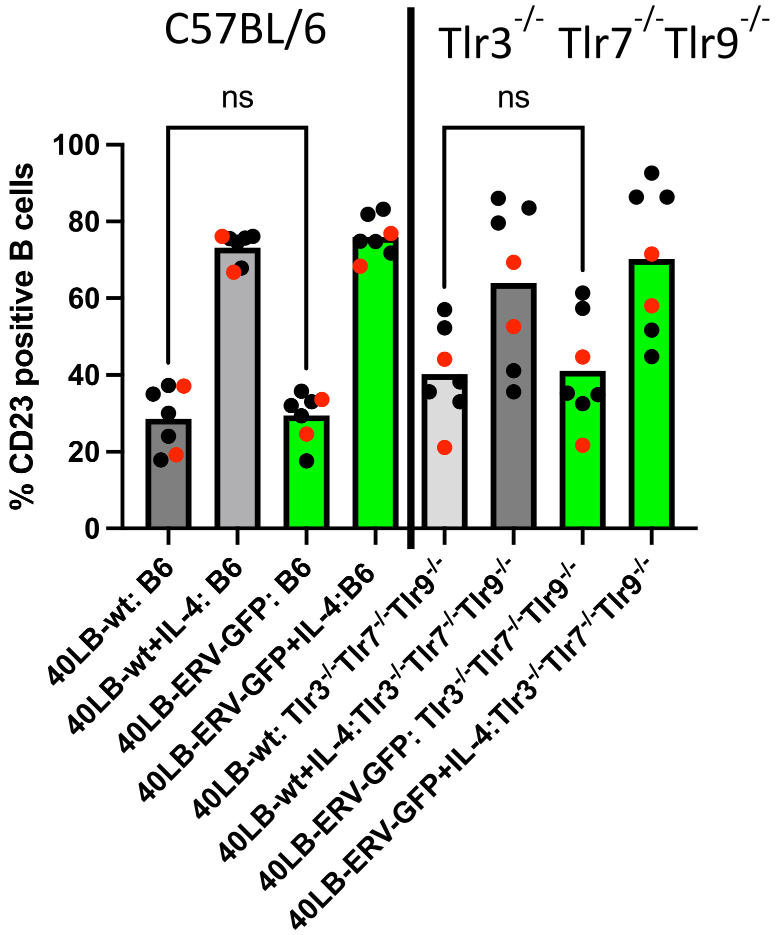

Figure S6. IgM-BCR induced B cell survival/proliferation in single Tlr7 and Tlr9 deficient B cells *in vitro* and CD23 induction by CD40L *in vitro.* Related to Figure 8.

a, Survival/proliferation of purified B cells from C57BL/6, Tlr3$^{-/-}$Tlr7$^{-/-}$Tlr9$^{-/-}$, Tlr7$^{-/-}$ and Tlr9$^{-/-}$ mice 72h after *in vitro* stimulation with either LPS, anti-IgM, polyI:C (Tlr3 ligand), R848 (Tlr7 ligand), 1668 PTO (Tlr9 ligand), and Pam3Cys (Tlr2 ligand). Percentage of B cells in gate (blue, FSC^{high}-PI^{low}) were analyzed with Tukey´s multiple comparisons test for anti-IgM: C57BL/6 (black bar, mean= 14.8, SD= 6.2, n= 8), Tlr3$^{-/-}$Tlr7$^{-/-}$Tlr9$^{-/-}$ (red bar, mean= 3.8, SD= 1.7, n= 8), Tlr7$^{-/-}$ (blue bar, mean= 7.5, SD= 4.1, n= 5) and Tlr9$^{-/-}$ (brown bar, mean= 22.6, SD= 2.2, n= 3). C57BL/6 vs. Tlr3$^{-/-}$Tlr7$^{-/-}$Tlr9$^{-/-}$ ****P<0.0001, C57BL/6 vs. Tlr7$^{-/-}$ *P=0.0372, C57BL/6 vs. Tlr9$^{-/-}$ ^{ns}P=0.9995. Summary of 12 experiments. **b,** 40LB or 40LB ERV-GFP does not lead to increased CD23 *expression* in Tlr3$^{-/-}$Tlr7$^{-/-}$Tlr9$^{-/-}$ B cells. Flowcytometry of B cells cocultured for 72h on

40LB with or without IL-4 or ERV-GFP (green coulmns) C57BL/6 and $Tlr3^{-/-}Tlr7^{-/-}Tlr9^{-/-}$ cells (black dots). EGT-315 B6 and EGT-315 $Tlr3^{-/-}Tlr7^{-/-}Tlr9^{-/-}$ (red dots). CD23 positive cells in % of totall B cells were measured. 40LB-wt:B6 (mean= 28.6,SD= 8.32, n=7), 40LB-wt+IL-4:B6 (mean= 73.2,SD= 4.07, n=7), 40LB-ERV-GFP:B6 (mean= 29.4 ,SD= 6.33, n=7), 40LB-ERV-GFP+IL-4:B6 (mean= 76.0,SD= 5.25, n=7), 40LB-wt:$Tlr3^{-/-}Tlr7^{-/-}Tlr9^{-/-}$ (mean= 40.2 ,SD= 12.1, n=7), 40LB-wt+IL-4: $Tlr3^{-/-}Tlr7^{-/-}Tlr9^{-/-}$ (mean= 64.0 ,SD= 20.8, n=7), 40LB-ERV-GFP: $Tlr3^{-/-}Tlr7^{-/-}Tlr9^{-/-}$(mean= 41.1 ,SD=14.2, n=7), 40LB-ERV-GFP+IL-4: $Tlr3^{-/-}Tlr7^{-/-}Tlr9^{-/-}$ (mean= 70.2 ,SD= 19.0, n=7). 40LB-wt:B6 vs 40LB-ERV-GFP:B6 ^{ns}P= >0.999. 40LB-wt:$Tlr3^{-/-}Tlr7^{-/-}Tlr9^{-/-}$ vs 40LB-ERV-GFP: $Tlr3^{-/-}Tlr7^{-/-}Tlr9^{-/-}$ ^{ns}P= >0.999. Tukey´s multiple comparisons test. Summary of 3 experiments. Source data are provided as a Source Data file.

Supplementary References

1. Beck-Engeser, G.B. *et al.* APOBEC3 enzymes restrict marginal zone B cells. *Eur J Immunol* **45**, 695-704 (2015).

2. Hemmi, H. *et al.* Small anti-viral compounds activate immune cells via the TLR7 MyD88-dependent signaling pathway. *Nat Immunol* **3**, 196-200 (2002).

3. Yu, P. *et al.* Nucleic acid-sensing Toll-like receptors are essential for the control of endogenous retrovirus viremia and ERV-induced tumors. *Immunity* **37**, 867-879 (2012).

4. Bauer, S. *et al.* Human TLR9 confers responsiveness to bacterial DNA via species-specific CpG motif recognition. *Proc Natl Acad Sci U S A* **98**, 9237-9242 (2001).

5. Rideout, W.M., 3rd *et al.* Generation of mice from wild-type and targeted ES cells by nuclear cloning. *Nat Genet* **24**, 109-110 (2000).

6. Sliva, K., Erlwein, O., Bittner, A. & Schnierle, B.S. Murine leukemia virus (MLV) replication monitored with fluorescent proteins. *Virol J* **1**, 14 (2004).

7. Majer, O., Liu, B., Kreuk, L.S.M., Krogan, N. & Barton, G.M. UNC93B1 recruits syntenin-1 to dampen TLR7 signalling and prevent autoimmunity. *Nature* **575**, 366-370 (2019).
