# Supplementary material for: T-bet+ B cells are activated by and control endogenous retroviruses through TLR-dependent mechanisms
Source: Nat Commun. 2024 Feb 9;15:1229. doi: 10.1038/s41467-024-45201-6 (PMC10858178; doi:10.1038/s41467-024-45201-6)
Supplement: Supplementary file 3 — Reporting Summary [file 41467_2024_45201_MOESM3_ESM.pdf]

nature portfolio

Corresponding author(s): Philipp Yu

Last updated by author(s): Dec 20, 2023

Reporting Summary

Nature Portfolio wishes to improve the reproducibility of the work that we publish. This form provides structure for consistency and transparency in reporting. For further information on Nature Portfolio policies, see our Editorial Policies and the Editorial Policy Checklist.

Statistics

For all statistical analyses, confirm that the following items are present in the figure legend, table legend, main text, or Methods section.

n/a	Confirmed	
☐	☒	The exact sample size (*n*) for each experimental group/condition, given as a discrete number and unit of measurement
☐	☒	A statement on whether measurements were taken from distinct samples or whether the same sample was measured repeatedly
☐	☒	The statistical test(s) used AND whether they are one- or two-sided<br>*Only common tests should be described solely by name; describe more complex techniques in the Methods section.*
☒	☐	A description of all covariates tested
☒	☐	A description of any assumptions or corrections, such as tests of normality and adjustment for multiple comparisons
☐	☒	A full description of the statistical parameters including central tendency (e.g. means) or other basic estimates (e.g. regression coefficient) AND variation (e.g. standard deviation) or associated estimates of uncertainty (e.g. confidence intervals)
☐	☒	For null hypothesis testing, the test statistic (e.g. *F*, *t*, *r*) with confidence intervals, effect sizes, degrees of freedom and *P* value noted<br>*Give P values as exact values whenever suitable.*
☒	☐	For Bayesian analysis, information on the choice of priors and Markov chain Monte Carlo settings
☒	☐	For hierarchical and complex designs, identification of the appropriate level for tests and full reporting of outcomes
☒	☐	Estimates of effect sizes (e.g. Cohen's *d*, Pearson's *r*), indicating how they were calculated

Our web collection on statistics for biologists contains articles on many of the points above.

Software and code

Policy information about availability of computer code

Data collection	Data collection for: 1. western blot analysis was done with the software included in the ChemiDoc XRS reader (BioRad). 2. ELISA was done with the Softmax Pro software of the Molecular Devices Emax Reader. 3. Flow cytometry used the CellQuest Pro software for data aquisition. 4. Confocal microscopy was done with the Leica LAS AF software for the TSC SP5 microscope. 5. ELISpot data were obtained by Eli.Scan (A.EL.VIS, Hannover, Germany) using the Eli.Analyse ELISPOT Analysis office Software V4.2. 6. For whole organ specific ERV-GFP expression we used the In Vivo Xtreme imaging system (Bruker Bio Spin, Hanau, Germany). Image acquisition as weil as analysis was performed using the Bruker MI SE software.
Data analysis	Data analysis was done for: l. western blot with software included in the ChemiDoc XRS reader (BioRad). 2.ELISA data was anlyzed with Softmax Pro software and GraphPad Prism. 3. Flow cytometry data was analyzed with FlowJo 8-10 software. 4. Confocal microscopy data was analyzed with Fiji ImageJ version 2.l.0/153c. 5. ELISpot data were analyzed by Eli.Analyse ELISPOT Analysis office Software V4.2.5. 6. In Vivo Xtreme imaging system (Bruker Bio Spin, Hanau, Germany) for organ ERV-GFP expression was used and image analysis was performed using the Bruker MI SE software. 7. For general data analysis, graphics and statistical analysis we used GraphPad Prism 9.5. 8. WGS-Sequencing data was analyzed with Cutadapt (v2.8), using segemehl (v0.3.4) and Trim Galore (v0.6.4).

For manuscripts utilizing custom algorithms or software that are central to the research but not yet described in published literature, software must be made available to editors and reviewers. We strongly encourage code deposition in a community repository (e.g. GitHub). See the Nature Portfolio guidelines for submitting code & software for further information.

Data

Policy information about availability of data

All manuscripts must include a data availability statement. This statement should provide the following information, where applicable:

- Accession codes, unique identifiers, or web links for publicly available datasets
- A description of any restrictions on data availability
- For clinical datasets or third party data, please ensure that the statement adheres to our policy

Sequencing data of WGS are deposited at the NCBI Sequence Read Archive (SRA) under BioSample accession SAMN38798973. Source data are provided with this paper. Any additional information required to reanalyze the data reported in this paper is available from the corresponding author.

Field-specific reporting

Please select the one below that is the best fit for your research. If you are not sure, read the appropriate sections before making your selection.

- [x] Life sciences
- [ ] Behavioural & social sciences
- [ ] Ecological, evolutionary & environmental sciences

For a reference copy of the document with all sections, see nature.com/documents/nr-reporting-summary-flat.pdf

Life sciences study design

All studies must disclose on these points even when the disclosure is negative.

Sample size	The sample size was not predetermined by statistical methods but rather based on intial results and availability of mice. All experiments were based on at least of 3 biological replicates and 3 independent experiments. The sample size or individual number of mice analyzed are stated in each figure legend.
Data exclusions	No data were excluded from analysis.
Replication	Individual mice of each genotype (n= l-2) were compared in individual experiments like Flowcytometry, immunhistology and in vitro stimulations. Data from replication experiments with identical conditions were then summarized as indicated in the figure legends giving the number of experiments and individual biological samples. The results of the experiments were reproducible
Randomization	The experiments were not formally randomized however selection of mice for experiments was done by technical staff solely selecting for match of age and sex of the experimental group.
Blinding	Formal blinding of the experiments was not applied. But for mouse experiments mice were given a number and subsequent data was acquired identical for all samples tested. Also analysis of data used objective standards for all samples like identical gates or settings thereby avoiding a bias. Also different individuals were performing experiments to further reduce a bias in data aquisition.

Reporting for specific materials, systems and methods

We require information from authors about some types of materials, experimental systems and methods used in many studies. Here, indicate whether each material, system or method listed is relevant to your study. If you are not sure if a list item applies to your research, read the appropriate section before selecting a response.

Materials & experimental systems

n/a	Involved in the study	
☐	☒	Antibodies
☐	☒	Eukaryotic cell lines
☒	☐	Palaeontology and archaeology
☐	☒	Animals and other organisms
☒	☐	Human research participants
☒	☐	Clinical data
☒	☐	Dual use research of concern

Methods

n/a	Involved in the study	
☒	☐	ChIP-seq
☐	☒	Flow cytometry
☒	☐	MRI-based neuroimaging

Antibodies

Antibodies used	Flow cytometry (antibodies used for Flow cytometry were diluted 1:300 for 1 million cells analyzed)<br>anti-CD4 PE (clone GKl.5 ;Thermo Fisher Scientific), anti-CD8a APC (clone 53-6.7; BD Biosciences), anti-Ly-6G PE (clone 1A8; Thermo Fisher Scientific), anti-CD45R/B220 APC (clone RA3-6B2; BD Biosciences), anti-CD23 PE (clone B3B4; BD Pharmingen), anti-CD23-Biotin (clone B3B4; Biolegend), anti-CDllc PE (clone N418; Thermo Fisher Scientific) and anti-CD90.2 APC (clone 53-2.1; BD Biosciences), anti-lgE-PE/FITC (clone 23G3, eBioscience); anti-CD20-Biotin (clone SA275All, Biolegend); anti-lgD-Biotin (clone

11-26c, Invitrogen); anti-IgD-FITC (clone 11-26c, Invitrogen); anti-IgM-APC (clone 11/41, BD Biosciences); anti-GL7-PE (clone GL-7, eBioscience); anti-CD19-PE (clone eBiolD3, eBioscience); anti-CD24-FITC (clone Ml/69, Invitrogen); anti-T-bet-e660 (clone eBio4Bl0, eBioscience). anti-CD80-PE (B7-19) eBiosciences, anti-CD73-APC (clone TY/11.8) BD Pharmingen. For counterstaining biotinilated Streptavidin-PerCP (Biolegend) was used. CDllc APC-cy7 (clone HL3) BD Bioscience 581241; CDllb BV 785 (Ml/70) Biolegend 101243; CD21 BV711 (7E9) Biolegend 123435; CD23 bio (B3B4) BD Bioscience 553137; GL7 perCP CyS.5 (GL7) Biolegend 144610; T-bet APC (4B10) Biolegend 644814; CD19 APC cy7 AS (6D5) Biolegend 115529; Strept BV570 Biolegend 405227
; Strept BV510 Biolegend 405234.
Western blot: Pcx Ab ThermoFischer PAS-72953
Immunhistochemistry
anti-CD23 PE (clone B3B4; BD Biosciences), anti-lgG (H+L) Alexa Fluor" 633 antibody (Thermo Fisher Scientific), anti-CD21/CD35 PE (clone 7E9; Miltenyi Biotee), anti-CD23 APC (clone B3B4, Thermo Fisher Scientific), anti-F4/80 PE (clone BM8; Thermo Fisher Scientific), and anti-CD4 APC (clone RM4-5; BD Biosciences), anti-lgG (H+L) Alexa Fluor" 633 antibody (Thermo Fisher Scientific). anti-CD4 PE (1:100, clone GKl.5; Thermo Fisher Scientific) and anti-CD45R/B220 APC antibody (1:100, clone RA3-6B2; BD, Biosciences).
Ca2+ mobilization
anti-IgM (STAR86, BioRad)
ELISA and ELISpot
We used anti-mouse-IgG (H+L) HRP (Jackson Immuno Research) as detection antibody for IgG specific ELISA and Elispot assays. For total IgE measurments anti-IgE (clone R35-72) was used to coat and for detection goat anti-IgE (Southern Biotech, HRP labeled) was used. As standard mouse IgE (clone MEA-36, Biolegend) was used.
For coat anti-mouse IL-6 (MAB406, R&D) and for detection biotin-rat anti-mouse IL-6 (BAF 406, R&D) were used.

Validation

1. anti-CD4 PE and anti-CD8a-APC were tested on wild-type thymus cells, staining resulted in the expected distribution of double postive and single postive thymocyte populations. 2. Ly-6G PE and anti-CDllc were positively validated on bone marrow cells and plasmacytoide dendritic cells derived in vitro (Flt3-culture) from bone marrow cells. 3. CD45R/B220 APC and anti-CD23 PE/Biotin antibodies were tested by comparing splenic B cells from C57BL/6 wildtype vs. CD23-deficient mice. Both antibodies identified B cells in wild type mice, but only CD45R/B220 APC positively stained B cells from CD23-deficient mice. We also validated anti-CD19-PE in this experiment. 4. CD90.2-APC was tested on Thy-1.2 T cells from the appropriate mouse strain. 5. anti-CD20 Biotin and anti-IgE-PE/FITC were successfully validated with IgE knock-in mice which express high levels of IgE bound on B cells. 6. anti-IgD-Biotin and anti-IgD-FITC , anti-IgM-APC, anti-GL7-PE were tested on spieen cells of immunized wild type mice. 7. T-bet-e660 was provided by Magada Huber (Medical Microbiology, Philipps-Universität Marburg) and validated in T cell experiments. 8. anti-IgM STAR86, BioRad was tested for its Ca2+ mobilization capacity on WEHI-231 B cells loaded with Fluo-4 indicator. 9. Antibodies for IgG and IgE ELISA and IgG Elispot were tested with serum from IgE knock-in mice and wild type mice. 10. Antibodies for coat anti-mouse IL-6 (MAB406, R&D) and for detection biotin-rat anti-mouse IL-6 were evaluated by testing IL-6 production from pDCs stimulated over night with various TLR-ligands. 11. In addition all antibodies are commercially available and have been tested by the companys which provide evaluation data. 12. The antibodies and streptavidin detection reagents for ABC cell analysis (Fig.5b and c):
CD11c APC-cy7 (clone HL3)
CD11b BV 785 (M1/70)
CD21 BV711 (7E9)
GL7 perCP Cy5.5 (GL7)
CD19 APC cy7 A5 (6D5)
Strept BV570
Strept BV510
were tested and evaluate by Thomas Winkler, Erlangen on autoimmune mice according to Nickerson et. al Age-associated B cells are heterogeneous and dynamic drivers of autoimmunity in mice. J Exp Med. 2023 May 1;220(5).
13. For Western Blot we used Pcx Ab ThermoFischer PAS-72953 (https://www.thermofisher.com/antibody/product/Pyruvate-Carboxylase-Antibody-Polyclonal/PA5-72953) and could confirm the antibody testing data provided.

Eukaryotic cell lines

Policy information about cell lines

Cell line source(s)

The WEHI-231 (ATCC CRL-1702) and NIH-3T3 cell line (ATCC CRL-1658) were from ATCC.The HEK-Blue™ hTLR7 Cells is from InvivoGen. HEK mTlr9 reporter cell line was made by Stefan Bauer, is described in the Methods references and can be obtained by request. 40LB was obtained from Daisuke Kitamura, University of Tokyo, Japan.

Authentication

All cell lines were frequently tested by flow cytometry (IgM expression of WEHI-231) and TLR-ligand stimulation and thereby authenticated. 40LB (NIH-3T3 with CD40L and BAFF) was tested by comparision to ist unmanipulated NIH-3T3 parent cell line, which was not able to support primary mouse B cell activation in vitro therefore confirming identity of 40LB.

Mycoplasma contamination

We use a HEK-hTLR2/CD14 reporter cell line assay applied every 4-6 weeks to test for mycoplasma contaminations of all cell lines used. All cell lines were negative.

Commonly misidentified lines
(See ICLAC register)

No commonly misidentified lines were used.

Animals and other organisms

Policy information about studies involving animals; ARRIVE guidelines recommended for reporting animal research

Laboratory animals

As experimental animals we used mice backcrossed to C57BL/6 background. As controls we used C57BL/6J and backcrossed the EGT-315 B6 and EZGT-332/3 B6 strains between 2-8 generations as indicated in figure legends, methods section and the manuscript

text. EGT-315 Tlr3-/-Tlr7-/-Tlr9-/- were backcrossed between 2-11 generations to Tlr3-/-Tlr7-/-Tlr9-/- on the C57BL/6 background (about > 18 generations backcrossed to C57BL/6). T-bet deficient mice on the C57BL/6 background were mated with EGT-315 B6 mice to obtain EGT-315 heterozygous mice with the T-bet deficiency. We also generated human Apobec3G (hA3) transgenic mice, which contain the human Apobec3 gene locus in this context: EGT-315 Tlr3-/-Tlr7-/-Tlr9-/- hA3; Tlr7-deficient mice and Tlr9-deficient single gene deficient mice which were used to isolate primary B cells for in vitro activation.

Mice for experiments were between 6 weeks and 10 month of age and age- and sex-matched for the experiments. Blood samples for screenings were taken at 3 weeks of age. For neonatal ERVGFP expression experiments mice were sacrificied between day 1 and day 7 after birth. Mice were housed under specific pathogenfree conditions in IVC cages with a 12h/12h light dark cycle, a humidity of 55%+/- 5% at a room temperature of 21oC+/- 1oC .

Wild animals	No wild animals were used for this study.
Field-collected samples	The study did not involve animals collected from the field.
Ethics oversight	The animal experiments were approved under V54-19c 20 15h 01 MR 20/8 Nr 84/2014 and V54-19c 20 15h 01 MR 20/8 Nr G63/2019 and Nr G80/2021 by the Regierungspräsidium Giessen, Hessen, Germany.

Note that full information on the approval of the study protocol must also be provided in the manuscript.

Flow Cytometry

Plots

Confirm that:

- [x] The axis labels state the marker and fluorochrome used (e.g. CD4-FITC).
- [x] The axis scales are clearly visible. Include numbers along axes only for bottom left plot of group (a 'group' is an analysis of identical markers).
- [x] All plots are contour plots with outliers or pseudocolor plots.
- [x] A numerical value for number of cells or percentage (with statistics) is provided.

Methodology

Sample preparation	By physical disruption we obtained single cell suspensions from spieen, bone marrow, lymph nodes and thymus. Cells were kept on ice in FACS buffer (PBS with 2% FCS+ 0.001% Azid). Red blood cell lysis was done with lysis buffer (8g NH4CI, lg KHCO3, 37.2 mg EDTA ad 11) for 8 min at RT. We used mouse lgG as Fe block (5 min at 4oC) and added a master mix of diluted antibodies to the samples (1 Mio cells in 100 microliter). After 30 min incubation at 4oC cells were washed and resuspend in FACS buffer (200 microliter) for acquisition.<br>For Ca2+ mobilization flow cytometry FACS buffer without azid was used. MACS purified B cells were loaded with Fluo-4 (2.5 micromolar) for 20 min at RT. After establishment of baseline Ca2+ concentration cells were stimulated by anti-lgM (25 microgram/ml in HBSS+ 2mM Ca2+, STAR86, BioRad) and measured for additional 4 min.
Instrument	FACSCalibur (Becton Dickinson), CytoFlex (Beckmann Coulter), CYTEK Aurora (CYTEK)
Software	For acquistion the BD software CellQuest Pro was used. Analysis was done with FlowJo software.
Cell population abundance	We did not employ flow cytometry sorting. For MACS bead (Miltenyi, Germany) based B cell enrichment, we used anti-CD43 beads to perform negative selection. CD43 negative naive splenic B cells were tested by anti-CD4 or anti-CD3e vs antiCD45RB/<br>B200 staining which ascertained between 93-98% purity of the B cell population.<br>ERV-GFP activation was examined by measurement of GFP expression in different cell populations. The expression varied between 0.001%-25% GFP positive cells depending on the genotype and age of the mice analyzed.<br>Other cell populations were identified at the expected ratios, with T-bet+ and GL7+ B cells being the exceptions because they were found increased to 1.75% and 0.757% respectively, only in EGT-315-B6 mice.
Gating strategy	For flow cytometry analysis we gated for lymphocytes and cells with higher granularity and size, excluding doublets, dead cells and residual red blood cells.<br>For flow cytometry of stimulated B cells (3 days with anti-lgM and various TLR-ligands) we used a gate that is characterized by blast phenoytpe (increased size by FSC) and negative for propidium iodide (live cells). This gate is shown in Fig. 56a and was also used for data of surviving/proliferating B cells in Fig 8d and 8e. Gating strategy for ABC cells iss layed out in Fig.5b.

- [x] Tick this box to confirm that a figure exemplifying the gating strategy is provided in the Supplementary Information.
